# Mental disorders in the Black population: a scoping review

**DOI:** 10.3389/fpubh.2026.1903601

**Published:** 2026-09-03

**Authors:** Rondinelli Salvador Silva, Yasmim Souza Rodrigues, Eduardo Sodré de Souza, Bruno Pereira da Silva, Luanda Ferreira Dias de Souza, Igor Hagedorn Reis, Ana Paula de Morais e Oliveira, Deivison Faustino Mendes, Patricia Lima Ferreira Santa Rosa

**Affiliations:** 1 School of Public Health, University of São Paulo, São Paulo, São Paulo, Brazil; 2 Institute of Psychiatry of the Medical School, University of São Paulo, São Paulo, São Paulo, Brazil; 3 School of Nursing, State University of Campinas, Campinas, São Paulo, Brazil; 4 Federal University of Acre, Cruzeiro do Sul, Brazil; 5 Faculty of Philosophy, Sciences and Letters at Ribeirão Preto, University of São Paulo, Ribeirão Preto, São Paulo, Brazil; 6 College of Nursing at Ribeirão Preto, University of São Paulo, Ribeirão Preto, São Paulo, Brazil; 7 School of Medical Sciences, State University of Campinas, Campinas, São Paulo, Brazil

**Keywords:** Black people, diversity, equity, inclusion, mental health, psychiatry, racism

## Abstract

**Introduction:**

Mental disorders, psychiatric diagnosis, DSM/ICD classification, and mental health care represent major sources of global morbidity shaped by biological processes, social stressors, structural racism, inequities, and access barriers. In the Black population, racism, discrimination, social exclusion, and institutional bias may affect recognition, diagnosis, treatment, prognosis, and psychiatric outcomes. This scoping review aimed to map and summarize the available evidence on mental disorders in the Black population worldwide, highlighting how psychiatric knowledge and mental health care are shaped by racialized social structures.

**Methods:**

The review was conducted according to JBI methodology and reported in accordance with the Preferred Reporting Items for Systematic Reviews extension for Scoping Reviews (PRISMA-ScR). Searches were conducted in MEDLINE, CINAHL, APA PsycINFO, Scopus, Embase, PubMed Central, Web of Science, the Virtual Health Library, the Brazilian Digital Library of Theses and Dissertations, and ProQuest Theses, with no restrictions on language or publication date. Sources addressing the Black population and mental disorders defined by diagnostic classification systems were included, covering manifestations, diagnoses, associated factors, access to care, interventions, barriers, and psychosocial consequences. Data were extracted using a purpose-built instrument and synthesized through thematic analysis, narrative synthesis, tables, and figures.

**Results:**

A total of 2,131 records were identified; after screening, search updating, and full-text assessment, 254 sources were included. Because 89.0% of included sources originated in the United States, the findings have limited transferability across diverse Black populations and healthcare contexts worldwide. Most sources examined clinical, hospital, outpatient, or healthcare settings, indicating that the mapped evidence primarily reflects formal care environments rather than community contexts. The most frequent disorders were depressive disorders, substance use disorders, psychosis or schizophrenia, anxiety, bipolar disorder, neurodevelopmental or conduct disorders, trauma, suicidality, and eating disorders. The main barriers and associated factors included gender, access to services, socioeconomic determinants, violence, trauma, racism, discrimination, and stigma.

**Conclusion:**

The evidence remains geographically concentrated and predominantly clinical-diagnostic, with limited transferability beyond the populations, services, classification systems, and contexts represented in included sources. These findings identify priorities for culturally responsive, antiracist, and equity-oriented research, but do not establish the effectiveness of specific policies, practices, or interventions.

**Systematic review registration:**

OSF registration doi: 10.17605/OSF.IO/WQAVD.

## 1 Introduction

Mental disorders (MDs) are defined as syndromes characterized by clinically significant disturbances in cognition, emotional regulation, or behavior, which often result from the complex interaction between biological processes and social stressors (1). In contemporary psychiatry, MDs constitute the main diagnostic unit, operationalized predominantly through classification systems such as the Diagnostic and Statistical Manual of Mental Disorders (DSM) and the International Classification of Diseases (ICD), despite persistent conceptual critiques (2, 3).

Moreover, MDs are highly prevalent worldwide, as one in eight people live with them (4). That is, nearly 1 billion people, corresponding to 14% of the world population, live with some psychiatric diagnosis (4). Thus, MDs are among the leading contributors to the global burden of disease (5).

Indeed, estimates indicate that MDs account for at least 18% of the global burden of disease, and their annual costs are expected to reach US$ 6 trillion by 2030 (6). Among these, anxiety disorders and mood disorders account for more than 50% of diagnoses, are more common among women and in developed countries, and are also a major risk factor for suicide (4).

Black populations may experience greater exposure to risk factors associated with racialized systemic inequalities, which may contribute to mental disorder burden across contexts (1, 7) These exposures vary across societies, reflecting differences in racial classification, socioeconomic conditions, migration histories, institutional arrangements, and unequal access to mental healthcare (8–10).

Throughout this review, Black is treated as a socially and historically produced racial category whose meanings vary across classification systems and contexts (8–10). It does not denote a homogeneous biological population, common ancestry, ethnicity, culture, or equivalent exposure to racialization, discrimination, and related social conditions (9, 10).

Accordingly, comparisons across studies were interpreted cautiously because self-identification, author-assigned categories, ancestry, nationality, and locally specific racial classifications are not interchangeable measures (8–10). This heterogeneity limits direct equivalence across populations and requires synthesis focused on contextual patterns rather than assuming a single universal Black population (8–10).

In this context, global reports of racism (11) against people of African descent are a cause for concern for the Office of the United Nations High Commissioner for Human Rights (11). This is because racism comprises a set of ideologies, attitudes, institutional practices, and social structures that generate and sustain racial inequalities, violating the fundamental principles of international law and undermining global peace and security (9).

Black populations have also reported less favorable experiences when accessing psychiatric care and treatment (7). Moreover, structural and interpersonal racism has been associated with mood, psychotic, and substance use disorders, potentially through chronic stress and social exclusion (1).

A meta-analysis of studies from Western countries reported a higher prevalence of depressive symptoms among Black participants than among White and Asian participants (12). The same meta-analysis estimated a 20.2% prevalence of depressive symptoms and found that high racial discrimination exposure was associated with greater symptom severity (12).

Despite this, evidence across the full range of mental disorders in Black populations remains limited, potentially constraining recognition of diagnostic and treatment biases (1). Race-related data remain incomplete, especially in low- and middle-income countries (4). Mapping this evidence may clarify reported associations between racism and mental disorders while supporting antiracist clinical tools and public health interventions (1, 12).

Available evidence is dispersed across diagnostic categories, clinical settings, social determinants, barriers, and interventions, limiting an integrated understanding of this heterogeneous field (4, 7, 12). Mapping its scope, characteristics, and gaps is therefore necessary to identify research concentrations, neglected contexts, and priorities for representative mental health research (13).

Thus, a preliminary search of the CDSR (Cochrane Database of Systematic Reviews), Epistemonikos, JBI Evidence Synthesis, MEDLINE (Medical Literature Analysis and Retrieval System Online), PROSPERO (International Prospective Register of Systematic Reviews), and OSF (Open Science Framework) databases revealed no records or systematic reviews of any type, completed or ongoing, focusing on the topic of MDs in the Black population.

Therefore, this scoping review aimed to map and summarize the available evidence on MDs in the Black population.

### 1.1 Review question

What evidence is available on the occurrence of MDs in the Black population worldwide?

## 2 Methods

The proposed scoping review was conducted according to the methodology proposed by the JBI (13) and registered on the Open Science Framework (OSF) platform (doi: 10.17605/OSF.IO/WQAVD.) The reporting of results followed the recommendations of the Preferred Reporting Items for Systematic Reviews and Meta-Analyses Extension for Scoping Reviews (PRISMA-ScR) (14) (Appendix A).

A scoping review systematically maps the breadth, characteristics, concepts, and gaps of heterogeneous evidence without restricting synthesis to narrowly defined outcomes or interventions (13, 14). Accordingly, this design accommodated diverse disorders, populations, settings, study designs, diagnostic systems, barriers, interventions, and psychosocial consequences relevant to the review question globally.

The objective was to characterize evidence rather than evaluate one intervention, compare narrowly defined outcomes, or calculate a pooled effect estimate across studies. For these reasons, a focused systematic review or meta-analysis would not answer the broader mapping question concerning classifications, contexts, barriers, and care dimensions.

This study used the following artificial intelligence (AI) resources: ChatGPT and Consensus. These resources assisted with bibliographic searches, writing refinement, and reference indexing. The illustrations were created using ChatGPT and Napkin. All processes involving Large Language Models (LLMs) and AI were conducted under human supervision and evaluation, in accordance with the principles of academic integrity.

### 2.1 Inclusion criteria

#### 2.1.1 Participants

Empirical studies whose participants were people from the Black population, either self-identified as such by the study participants themselves or classified as Black by the study authors, were included. Study participants were considered regardless of their personal, family, and social characteristics, such as age, gender identity, educational level, family membership, employment status, personal and/or family income, residence in their country of birth or immigrant status, and whether or not they had health insurance and social security.

In this scoping review, participants were considered Black when they self-identified as Black or were explicitly classified as Black by the study authors (15).

Evidence produced without an explicit ethnic-racial focus on the Black population, or evidence addressing mental health in generic terms without allowing the identification of data or analyses specific to this population, was excluded.

Participants were included across ages, genders, socioeconomic positions, migration statuses, and insurance conditions because the review sought comprehensive mapping rather than subgroup restriction. An explicit Black population focus was required so findings could be attributed specifically to this population instead of inferred from racially aggregated samples.

#### 2.1.2 Concept

The definitions of MDs presented in the various versions of the DSM and/or ICD were used for this scoping review. Evidence addressing clinical manifestations, psychiatric diagnoses made by medical and non-medical professionals, associated factors, access to care, health interventions of any kind, structural or social barriers faced, and the psychosocial consequences of MDs, as defined according to DSM and/or ICD versions, in the Black population was considered for inclusion. Diagnoses established by medical and non-medical clinicians were eligible because diagnostic responsibilities vary internationally across professions, service models, and different health systems (16).

Neurocognitive disorders alone were excluded because their inclusion would broaden the review beyond its psychiatric focus and introduce distinct diagnostic and etiological considerations. Studies were eligible only when a clinician formally established a mental disorder according to DSM or ICD criteria, regardless of concurrent instrument use. Excluding instrument-only classifications increased diagnostic comparability but may have reduced coverage where clinician-based assessment was unavailable, inaccessible, or not routinely documented in practice.

Clinical manifestations, associated factors, access, interventions, barriers, and psychosocial consequences were included to map how diagnosed disorders were experienced and managed across contexts. DSM or ICD classifications were required to maintain a consistent operational boundary for mental disorders across countries, periods, professions, and major diagnostic traditions.

#### 2.1.3 Context

All studies were considered for inclusion regardless of the geographical location of the country of origin, the culture in which they were developed, the type of health system in place (public, private, or mixed), or whether they were developed by nongovernmental organizations, in inpatient or outpatient settings, or in religious, philanthropic, and similar institutions.

All geographical, cultural, institutional, and healthcare contexts were eligible because the review aimed to map evidence globally and examine variation across service arrangements. No contextual exclusions were applied because restricting settings or health systems could obscure underrepresented evidence and reduce understanding of how classifications operate internationally.

#### 2.1.4 Types of sources

This scoping review considered both experimental and quasi-experimental study designs, including randomized clinical trials, nonrandomized clinical trials, before-and-after studies, and interrupted time-series studies. In addition, analytical observational studies, including prospective and retrospective cohort studies, case–control studies, and analytical cross-sectional studies, were considered for inclusion. This scoping review also considered descriptive observational study designs, including case series, individual case reports, and descriptive cross-sectional studies.

Qualitative studies focusing on qualitative data were also considered, including, but not limited to, designs such as phenomenology, grounded theory, ethnography, qualitative description, action research, and feminist research. In addition, systematic reviews that met the inclusion criteria were also considered. With regard to gray literature, theses and dissertations were included.

Experimental, observational, qualitative, and evidence-synthesis designs were eligible because each could contribute distinct information about diagnosis, experiences, services, barriers, interventions, or relevant outcomes. Theses and dissertations were included to capture gray literature, reduce dependence on journal publication, and broaden coverage of emerging or locally produced evidence.

### 2.2 Search strategy

APMO, a librarian at the University of Campinas, has extensive experience supporting literature searches on Black populations and racial issues in health research. She collaborated throughout strategy development, conducted searches with the authors in real time, organized records in Rayyan, and contributed to initial study conceptualization. This procedure followed the three stages recommended by the selected method: first, a preliminary search was conducted in MEDLINE (via PubMed) and CINAHL (via EBSCO) to identify relevant articles on the topic. The databases, keywords, and descriptors extracted from the titles, abstracts, and indexing terms of the identified articles were used to build a comprehensive search strategy.

The initial search was conducted in April 2025 and updated in February 2026, without restrictions on language or publication date at either stage. Thus, the 1983–2026 interval emerged empirically from eligible publication years, while retrospective data reaching the 1970s did not alter publication-date eligibility for this review. Works produced in languages other than Portuguese or English were translated using DeepL Translate (17).

### 2.3 Information sources

The databases searched were MEDLINE (via PubMed), CINAHL (via EBSCO), APA PsycINFO, Scopus (Elsevier), Embase, PubMed, PMC, Scopus, Web of Science, and the Virtual Health Library (VHL). For the gray literature search, the Brazilian Digital Library of Theses and Dissertations (BDTD) and ProQuest Theses were searched. The reference lists of all included studies were manually examined to identify additional potentially relevant studies not identified during the database search stage.

### 2.4 Study/source of evidence selection

All identified citations were imported into Zotero (Zotero 9.0.3), then uploaded to Rayyan (Qatar Computing Research Institute, Doha, Qatar), and duplicates were removed. After this stage, a pilot test was applied so that reviewers RSS and YSR could independently screen titles and abstracts based on the eligibility criteria. Potentially relevant sources had their full texts retrieved and fully assessed by the same two independent reviewers. Any discrepancies were resolved by consensus or with the collaboration of a third reviewer, PLFSR.

**Figure 1:**
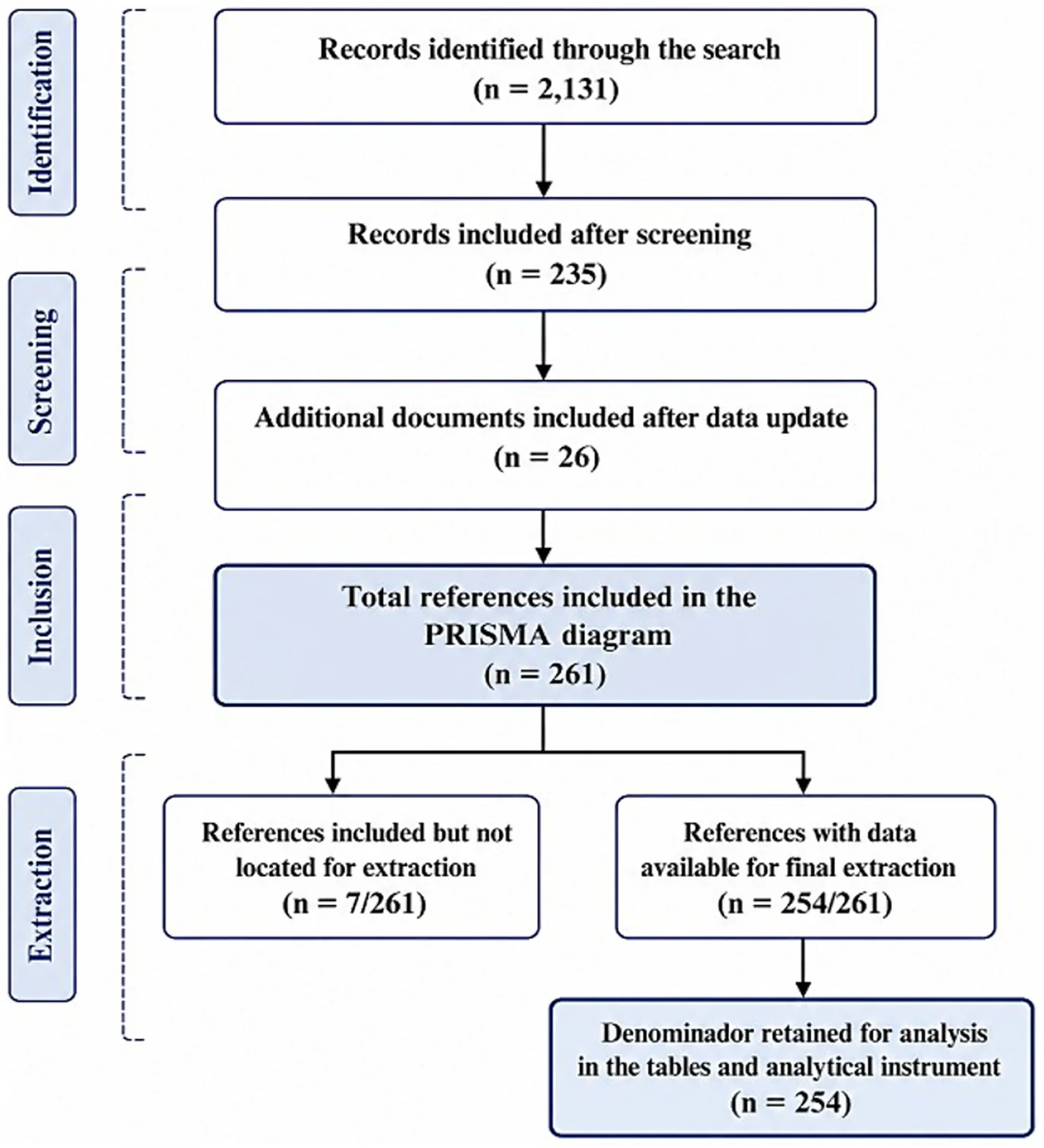
PRISMA flow diagram showing the selection and extraction process of the studies included in this scoping review.

As a result of the search, 2,131 records were identified, of which 235 were selected after title and abstract screening. Subsequently, in the second search conducted, an additional 26 records were included, totaling 261 results. Seven included references could not be located, even after contacting the authors. Thus, 254 records remained and were subjected to full-text reading, followed by the data extraction process. The selection process was reported in detail using the PRISMA-ScR flow diagram (Figure 1).

### 2.5 Data extraction

Data were extracted by two independent reviewers, RSS and YSR, using a tool developed specifically for this review (Appendix C). The extracted information included details on participants, such as age group, gender, and racial criteria; concept, including type of mental disorder, diagnostic categories, and associated factors; context, including country, health system, and social markers; methodological design; and main findings. The data extraction instrument was initially applied to a 10% sample, in which its feasibility or need for adjustments was assessed, considering the review question and objective.

### 2.6 Data analysis and presentation

Data analysis used thematic analysis in a descriptive sense, as prespecified in the protocol, rather than following a named framework or author-specific procedure. Familiarization involved repeated reading of extracted findings before initial codes were generated inductively from recurring content relevant to the review question and objectives.

Initial coding of included sources was divided equally among IHR, LFDS, RSS, and YSR using extracted findings recorded in the shared Excel spreadsheet. Each researcher subsequently reviewed all codes and supporting excerpts produced by the others, enabling reciprocal verification across the complete coded dataset before synthesis.

A coding framework was maintained in Excel and updated iteratively as recurrent codes were compared, clarified, merged, separated, or retained during reciprocal review. Disagreements concerning code application or interpretation were discussed among the four coders and PLFSR until consensus was reached and the framework was refined.

Recurrent codes were jointly grouped into candidate themes, which were reviewed for internal coherence, conceptual distinctiveness, relevance, and consistency with the extracted findings. Theme names, boundaries, and contents were then defined collaboratively before the final narrative synthesis, tables, figures, Results, and Discussion sections were collectively prepared.

### 2.7 Critical appraisal

Critical appraisal was not conducted because this review mapped the extent, characteristics, and gaps of heterogeneous evidence rather than quantitatively estimating effects (13). Consequently, findings describe patterns across sources without weighting methodological rigor and should not be interpreted as evidence of effectiveness, causality, or overall certainty.

## 3 Results

Detailed extraction results are presented in Appendix D; unless otherwise stated, percentages represent proportions of the 254 sources containing each relevant coded category. Categories could overlap when one source contributed to multiple coded categories; therefore, percentages within several groups were not necessarily expected to total 100%.

**Figure 2:**
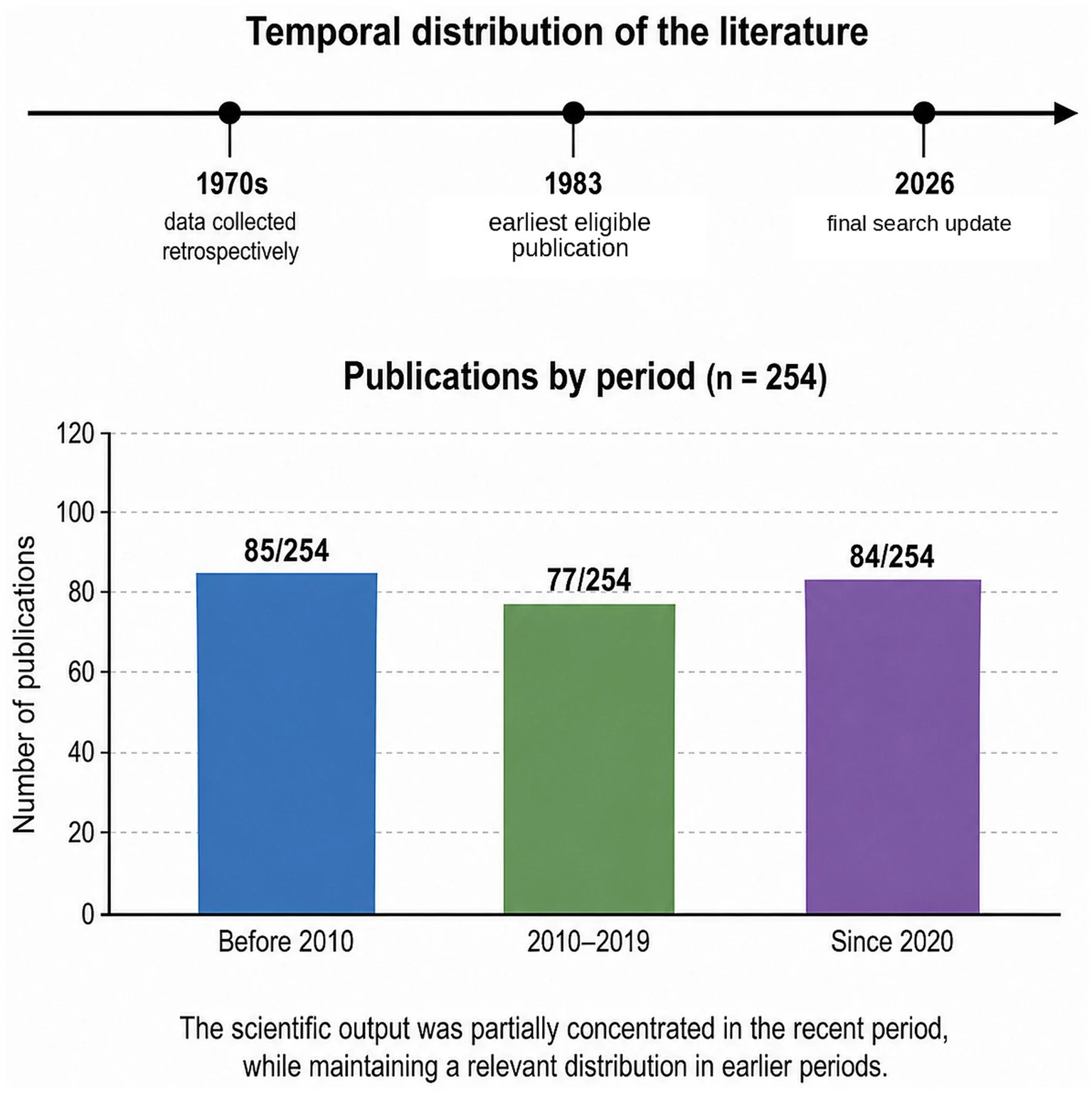
Temporal distribution of the studies included in the scoping review.

Eligible publications ranged from 1983 to 2026, while two included sources reported retrospectively collected data extending into the 1970s within reviewed populations (18, 19). Publication year was available for 246 sources: 85 (33.5%) appeared before 2010, 77 (30.3%) during 2010–2019, and 84 (33.1%) from 2020 onward overall (Figure 2).

**Figure 3:**
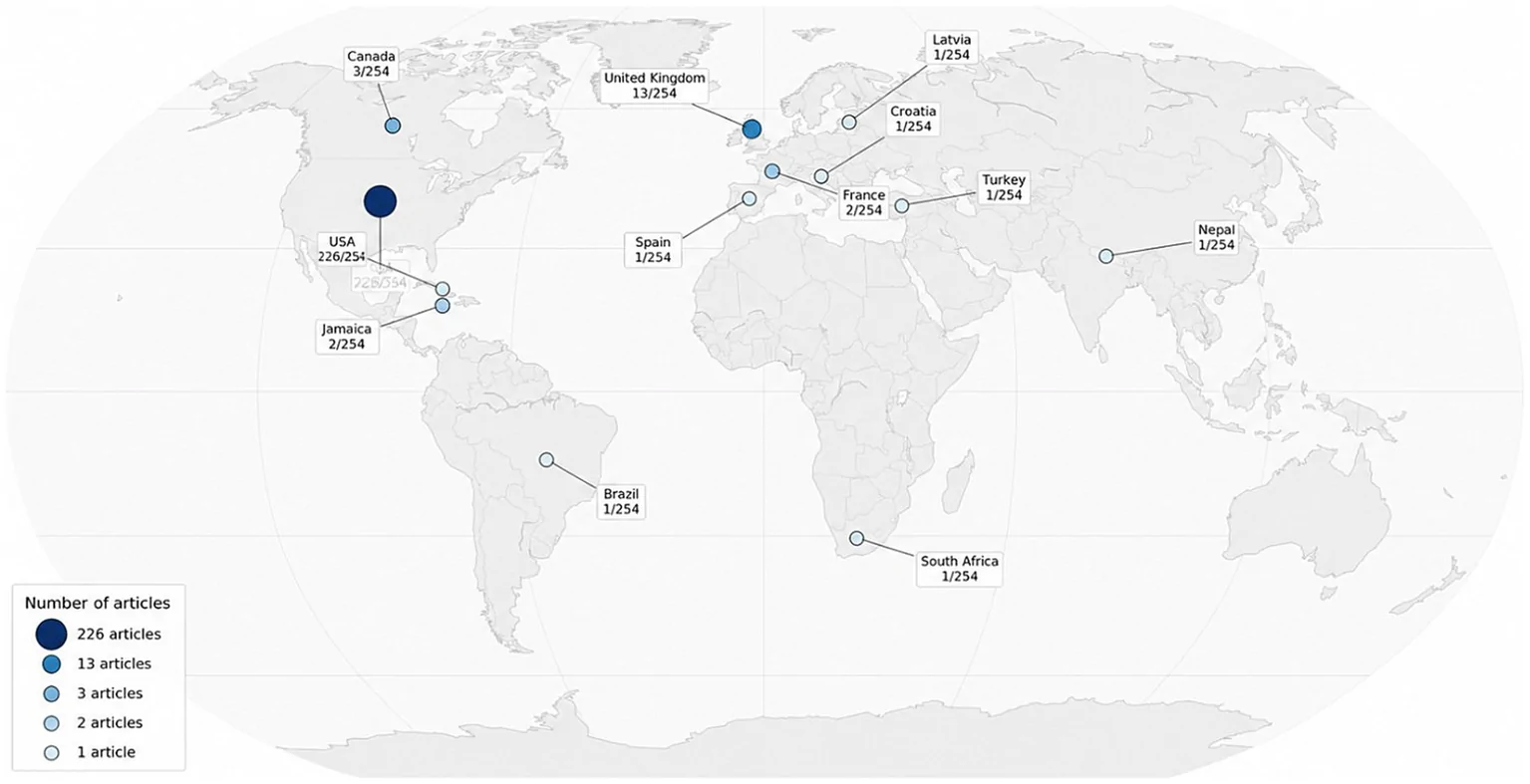
Distribution of scientific production included in the scoping review.

There was a predominance of publications from the United States, with 89.0%, followed by the United Kingdom with 5.1%, while Canada, Jamaica, and France comprised 1.2, 0.8, and 0.8%, respectively, of the predominant geographic basis currently reviewed (Figure 3). Brazil, South Africa, Turkey, Nepal, Cuba, Croatia, Latvia, and Spain had lower production, corresponding to 3.1%.

**Figure 4:**
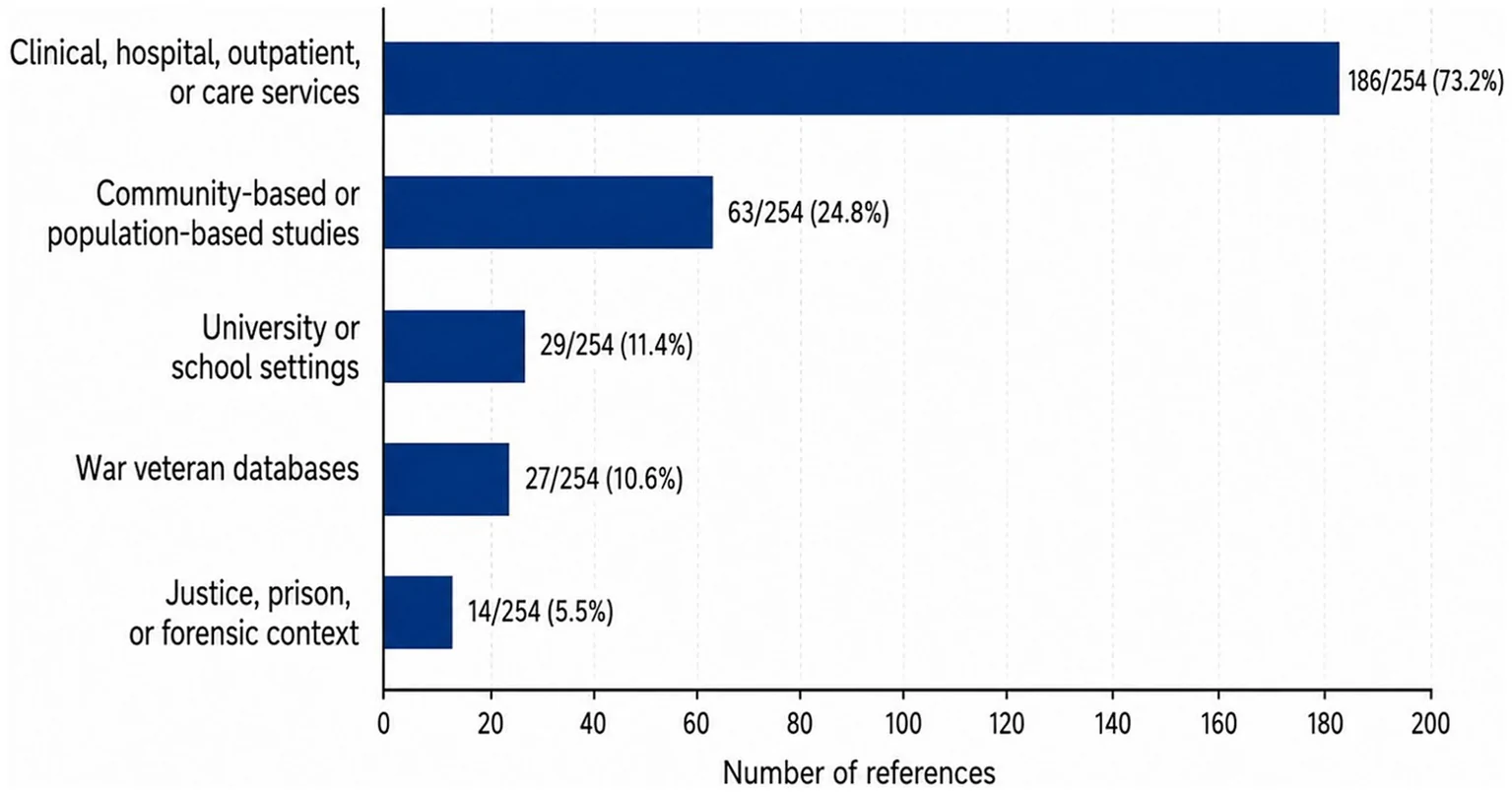
Number of references according to the contexts in which the studies were conducted.

Clinical, hospital, outpatient, or healthcare services appeared in 73.2% of references. Community or population-based studies appeared in 24.8% of the references; university or school settings in 11.4%; war veteran databases in 10.6%; and justice, prison, or forensic contexts in 5.5% (Figure 4).

**Figure 5:**
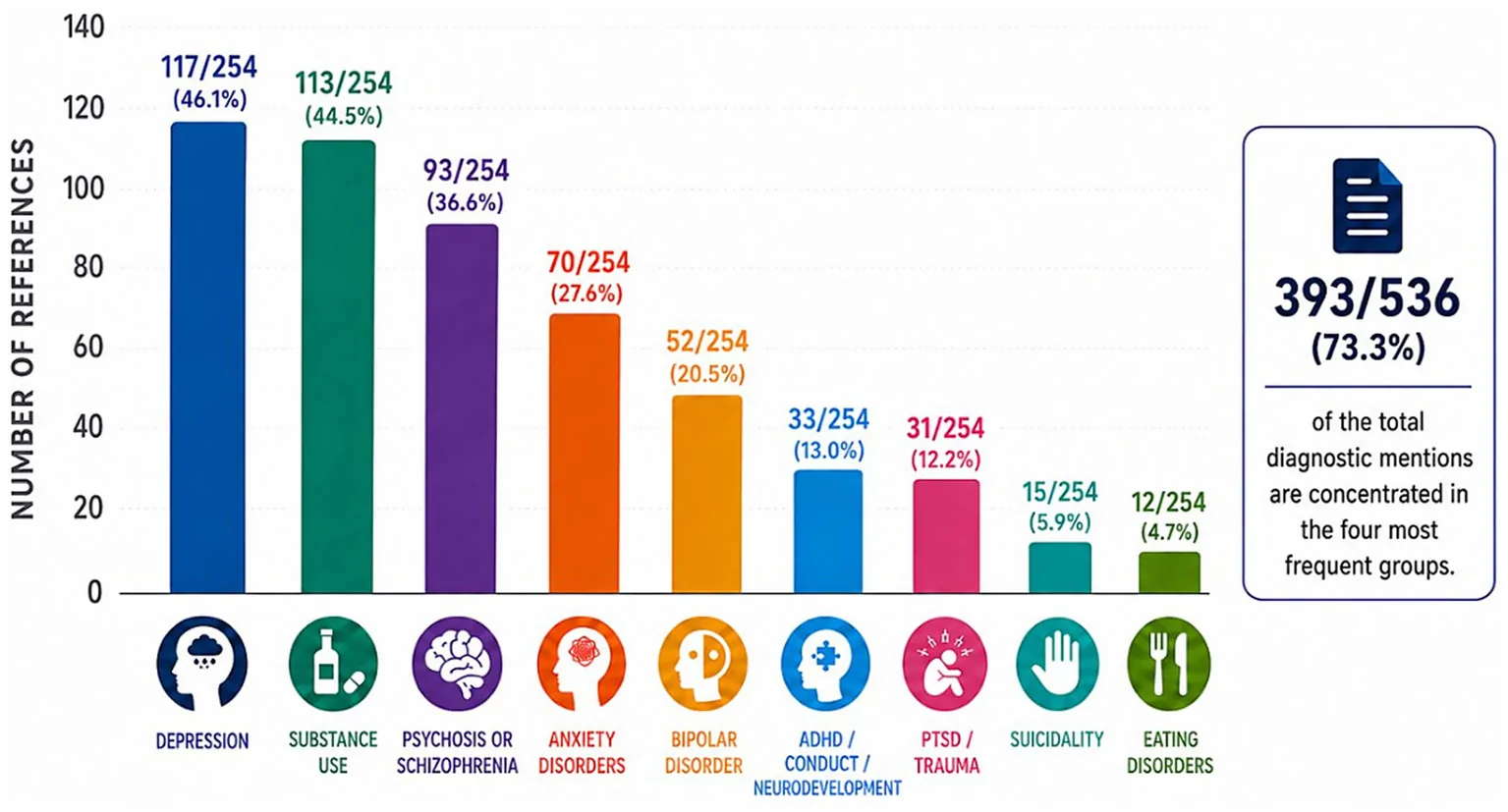
Distribution of mental disorders by number of references.

Depressive disorders were the most recurrent, present in 46.1% of references, followed by substance use disorders in 44.5% and psychosis or schizophrenia in 36.6%, in the diagnostic field. Anxiety disorders appeared in 27.6%, bipolar disorder in 20.5%, attention-deficit/hyperactivity disorder (ADHD), conduct disorder, or neurodevelopmental disorders in 13.0%, PTSD or trauma in 12.2%, suicidality in 5.9%, and eating disorders in 4.7% (Figure 5).

**Figure 6:**
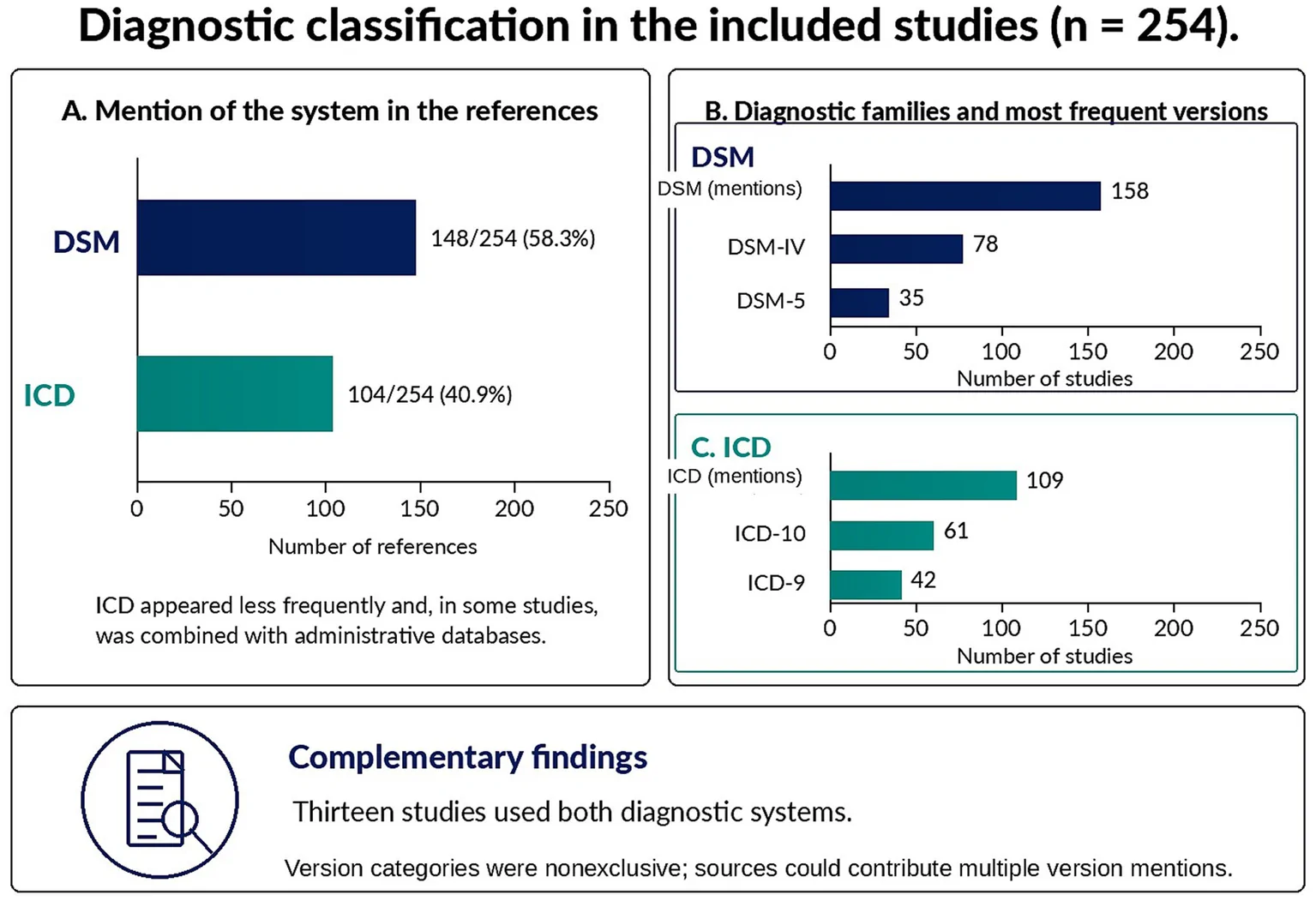
Distribution of the number of references according to diagnostic category: **(A)** Mention of the system in the references (DSM and ICD); **(B)** Diagnostic families and most frequent versions (DSM-IV and DSM-5); **(C)** Diagnostic Families and Most Frequent Versions (ICD-10 and ICD-9).

DSM classifications appeared in 148 sources (58.3%), whereas ICD classifications appeared in 104 sources (40.9%); 13 included sources used both diagnostic systems concurrently. Because sources could report multiple editions, coding yielded 158 DSM-version mentions and 109 ICD-version mentions, including DSM-IV, DSM-5, ICD-10, and ICD-9 across studies (Figure 6).

**Figure 7:**
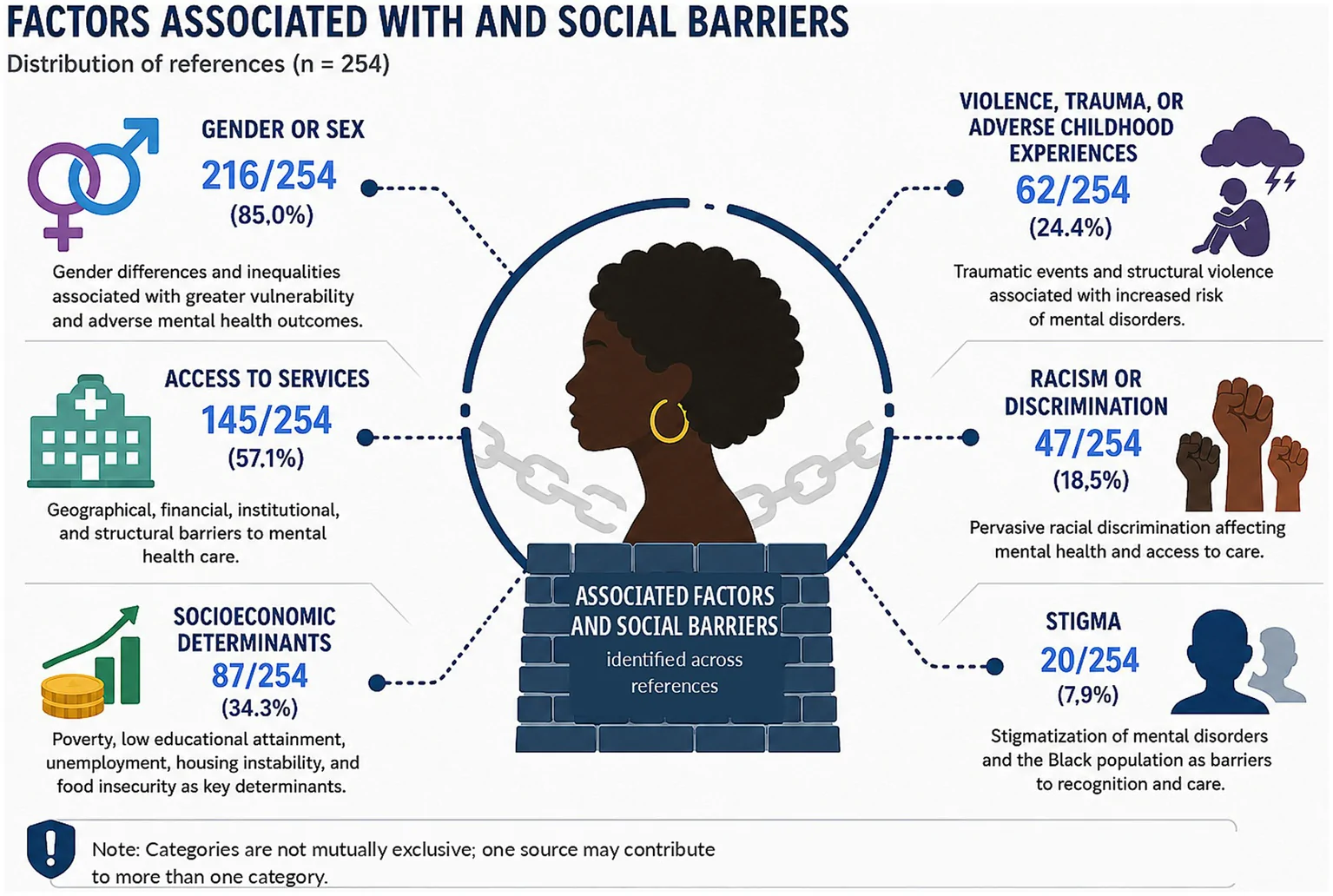
Associated factors and social barriers found in the references included in this scoping review.

Associated factors and barriers highlighted gender or sex in 85.0% of references, access to services in 57.1%, and socioeconomic determinants in 34.3%, as recorded in the fields on social factors and barriers. Violence, trauma, or adverse childhood experiences appeared in 24.4% of references; racism or discrimination in 18.5%; and stigma in 7.9% (Figure 7).

**Figure 8:**
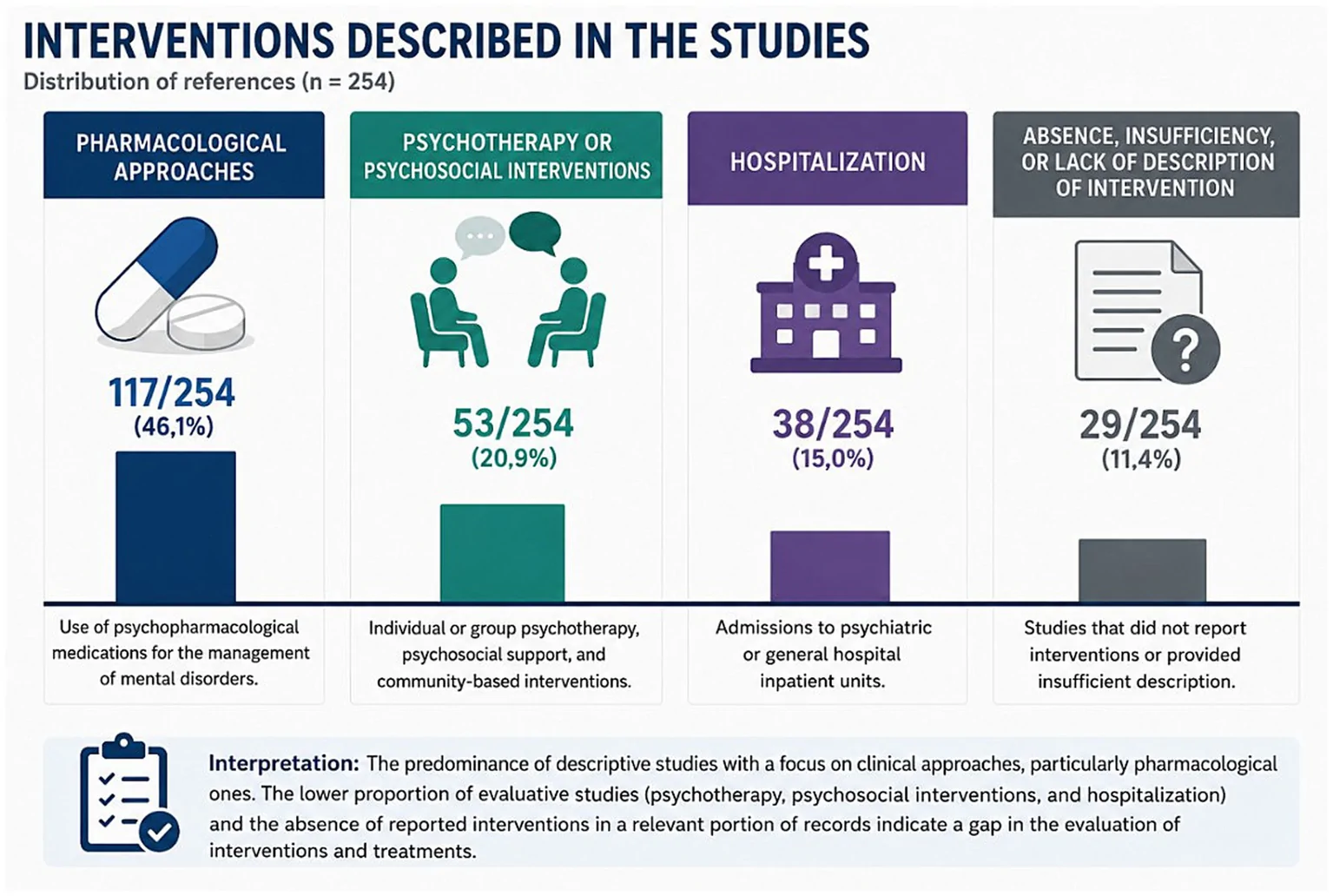
Interventions described in the studies included in this scoping review.

The interventions described were predominantly clinical: pharmacological approaches appeared in 46.1% of references, psychotherapy or psychosocial actions in 20.9%, and hospitalization in 15.0%, in the specific field on extracted actions and treatments. In 11.4% of references, interventions were absent, insufficiently described, or not reported (Figure 8).

**Figure 9:**
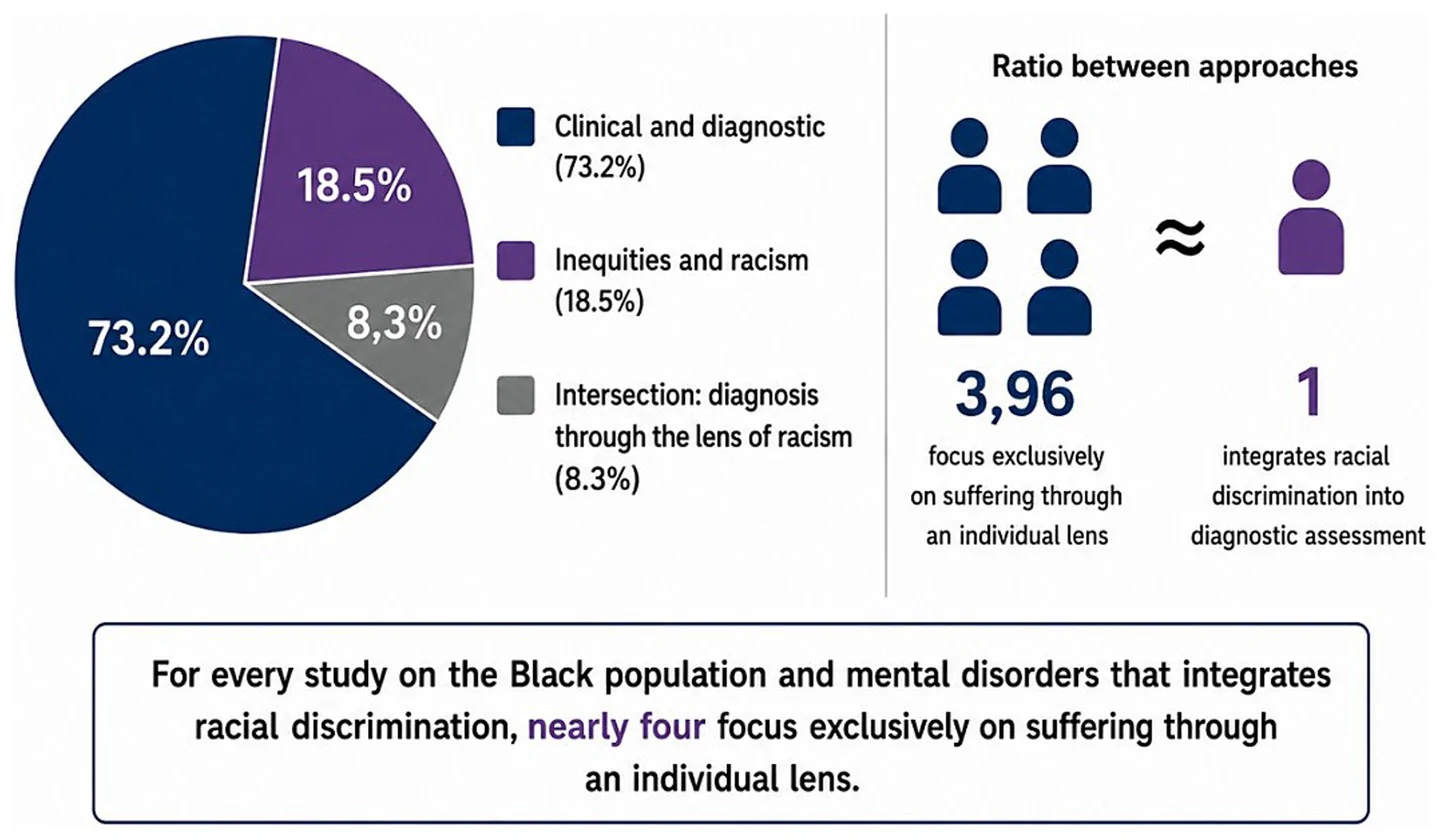
Approaches in the literature on mental disorders in the Black population.

References were classified as clinical-diagnostic, focused on inequities and racism, or integrating diagnosis with racism, representing 73.2, 18.5, and 8.3% of sources, respectively. These categories summarize analytical emphases assigned during coding and should be interpreted as source frequencies rather than population prevalence or comparative effect estimates (Figure 9).

## 4 Discussion

Because no critical appraisal was conducted, the mapped patterns cannot establish certainty or methodological robustness of claims concerning diagnostic bias and structural racism. Interpretations therefore reflect recurring findings across heterogeneous sources and should not be understood as universal conclusions across all Black populations, settings, or contexts.

### 4.1 Nosological alienation and the petrification of diagnosis

The mapped literature was predominantly clinical and diagnostic, while fewer sources addressed racism, inequities, or their intersections with psychiatric classification and mental healthcare. Accordingly, the Discussion proceeds from descriptive patterns toward cautious interpretation of diagnosis, social determinants, treatment, institutional barriers, and geographical concentration across the evidence.

Frantz Fanon describes alienation as the process through which the colonized subject internalizes the inferiority imposed by the clinical gaze that pathologizes their existential relations within an oppressive structure (20). Schizophrenia overdiagnosis was discussed in 9.8% of mapped sources, suggesting that some diagnostic practices may insufficiently account for social determinants and institutional racism (21).

In 7.1% of sources, authors described difficulties interpreting culturally specific distress, including affective presentations classified as severe psychotic symptoms among Black men (22, 23). These findings suggest that diagnostic categories may insufficiently incorporate racialized histories and cultural context, although this review cannot reliably determine causal relationships (21).

Some included sources described cultural distrust toward health systems as being interpreted clinically as paranoia, rather than considered in relation to histories of recurrent violence (24). Hence the relevance of a psychiatric practice that recognizes structural racism, institutional biases (25), and cultural misunderstanding in diagnostic formulation, avoiding interpretations that deepen inequalities and marginalize Black patients in contemporary care (26).

This argument is reinforced by evidence that diagnostic rates for mood disorders remain significantly lower among Black individuals compared with White individuals, reinforcing the clinical bias of greater attributed pathological chronicity (11.0%). This is illustrated by persistent racial barriers that reduce the recognition of and access to adequate clinical, specialized, and pharmacological care for Black people with major depression (27) in contemporary mental health care (28).

### 4.2 The sociogeny of suffering and racial etiology

Building on these diagnostic patterns, 13.8% of sources associated perceived racism with severe depressive symptoms among Black adolescents, without establishing causal direction (29). Because most contributing evidence was observational, these relationships may also reflect socioeconomic conditions, trauma exposure, service access, and other measured or unmeasured factors.

Across mapped sources, adverse childhood experiences, community violence, and discrimination were associated with post-traumatic stress symptoms and poorer health-related outcomes among Black participants. Socioeconomic disadvantage and restricted service access were also frequently reported, limiting attribution of disparities to any single exposure or pathway across heterogeneous settings.

In 16.5% of the results, it was shown that minority stress and continuous exposure to structural racism may produce psychobiological wear (30), involving neuroendocrine activation (31), alterations in the HPA axis (32), and increased inflammatory markers (33). In this specific context, the mental suffering of the Black population is a psychosocial expression of structural racism, which produces trauma, chronic stress, and institutional inequalities capable of simultaneously affecting the body, subjectivity, and care (25).

Ignoring the political dimension of racialized illness reduces medicine to symptomatic management, medicating effects while preserving racist structures that produce persistent mental suffering in Black subjects and social illness (7). This is the case of the impact of the COVID-19 pandemic, which exacerbated these depressive symptoms in Black men (34), revealing the extreme fragility of current social and community support systems (19.7%).

### 4.3 Zones of nonbeing and inequities in pharmacological treatment

These sociogenic patterns also extended to treatment, where 20.5% of sources described restricted access to advanced pharmacological care among included Black patients (29). In 21.7%, Black patients experiencing opioid overdose received fewer buprenorphine prescriptions than White patients, indicating unequal treatment across diverse mapped clinical care settings.

In 22.8% of sources, first-generation antipsychotic use was reported among older Black adults, while 23.6% described incomplete documentation of adverse effects (Appendix D). These patterns may indicate unequal treatment or monitoring, but the mapped evidence cannot establish that race independently caused prescribing decisions or undocumented harms.

**Figure 10:**
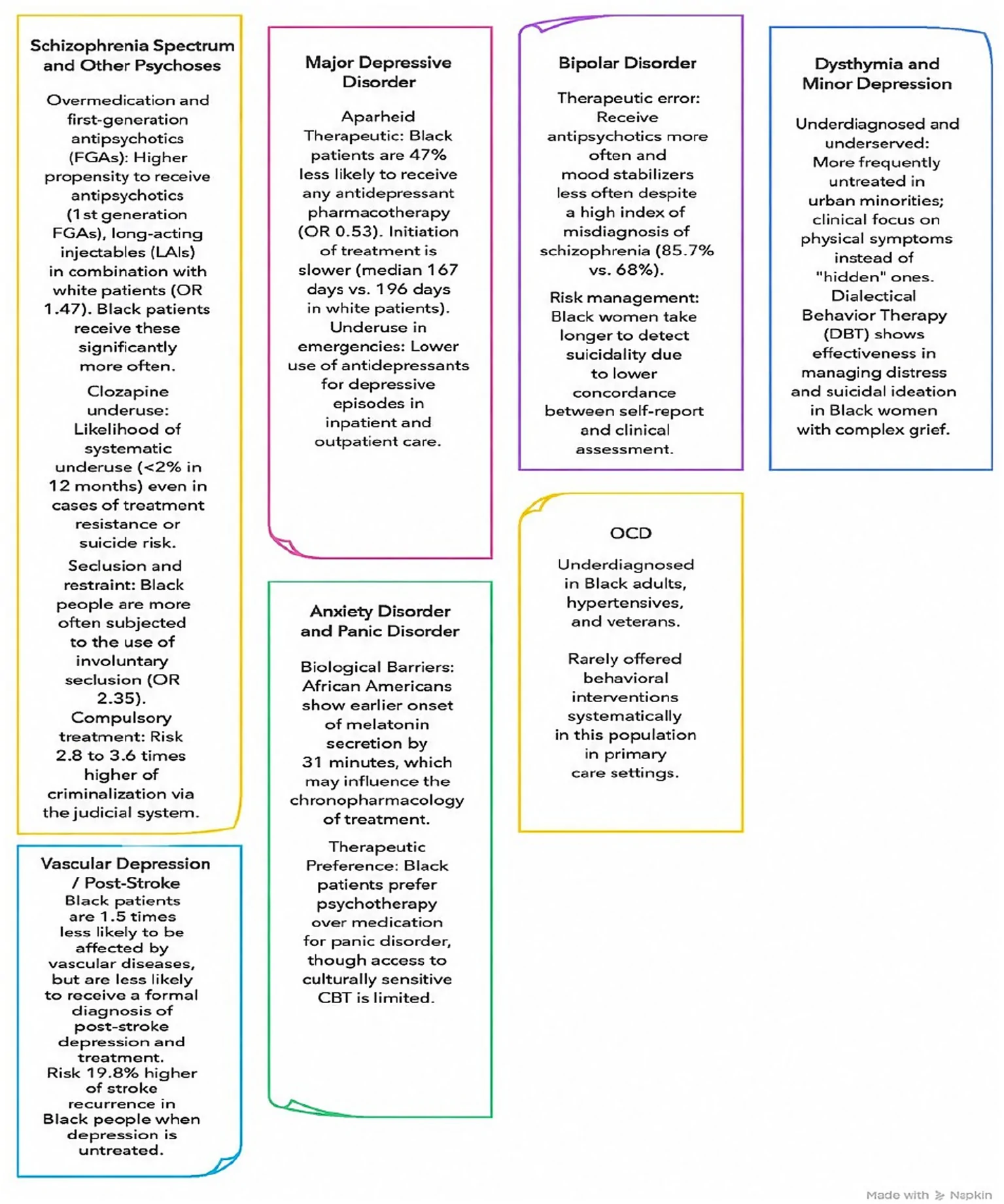
Racial inequities in the psychiatric treatment of the Black population.

Disparities in the treatment of post-stroke depression, observed in 24.4% of the evidence, indicate that Black patients face barriers that go beyond financial access, involving implicit bias among health professionals and insufficient systematic assessments to identify depressive symptoms. In 25.6% of the studies, there was underutilization of specific medications for alcohol use disorder in intensive care units, reinforcing the cycle of neglect toward the most vulnerable populations (Figure 10).

In 26.8% of the results, a clinical preference for disciplinary interventions was found, including involuntary seclusion, compulsory and coercive hospitalizations, admission to prisons and forensic units, school punishment instead of clinical assessment, and coercive use of medications (Appendix D), to the detriment of modern therapeutic approaches when the patient belongs to historically marginalized racialized or ethnic groups. Democratizing access to biotechnological innovations is a fundamental step toward ensuring that human dignity is not a privilege reserved only for those who possess recognized social whiteness (35).

### 4.4 Structural barriers and institutional control of the body

Beyond treatment inequalities, institutional barriers to early autism and ADHD diagnosis were reported in 28.3% of sources involving Black children across educational settings. Black boys received fewer psychostimulant prescriptions than White boys, suggesting behavioral interpretation and disciplinary responses may shape access to appropriate clinical support (36, 37).

Across 30.7% of sources, prolonged waiting lists and denial of government benefits were described alongside structural barriers affecting equitable access to mental health services (38, 39). These systemic inequities operate as mechanisms of exclusion that perpetuate the social marginality of Black subjects with special needs who find no support within state protection networks (40).

A hospitalization and admission rate 4.06–4.63 times higher for first-episode psychosis among Black African and Caribbean people in the United Kingdom was observed in 8.3% of the articles. In 7.5% of the results, post-traumatic stress disorder was almost twice as frequent among African Americans, with a rate of 8.7%, in addition to inequalities in the recognition and follow-up of ADHD, including underprescription in 3.9% of the articles, criminalization of Black adolescents (41–45), and autism spectrum disorder, including racial inequity in consultations (46–51) and denial of benefits (47, 50, 52) throughout clinical care.

Diagnostic barriers, present in 7.5% of the results, include psychotic readings of mood disorders, diagnostic error involving bipolar disorder and schizophrenia in 85.7% of Black people undergoing treatment, increased risk of major depressive disorder due to racism (51), underdiagnosis, and stigmatizing labels, including malingering (53–55) and tardive dyskinesia (53, 55–59). In treatment, there was 47% lower pharmacological adequacy for major depressive disorder in 7.1% of the results, greater use of long-acting injectable antipsychotics and high doses in 5.9%, less use of clozapine (60–67), psychostimulants (42, 45, 46, 48, 49, 68–72), buprenorphine (73–80), naltrexone and acamprosate (76, 81–88), as well as lower community support (47, 50, 52), higher prevalence of police coercion (43, 89–91), and persistent systemic inequities (52, 73, 92–95).

**Figure 11:**
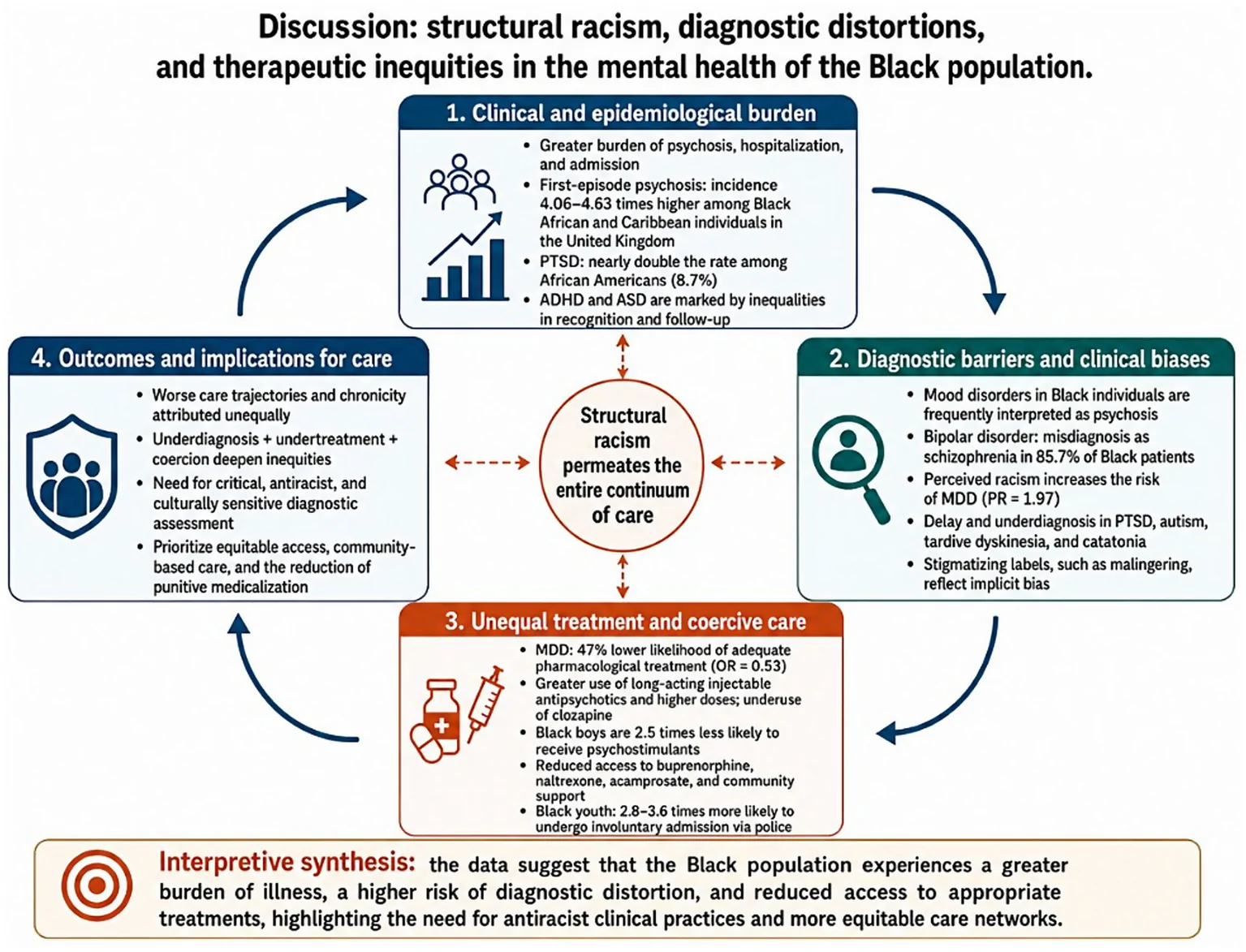
A cyclical model diagram.

Figure 11 schematizes structural racism at the center and its interrelationship with four components: clinical and epidemiological burden, diagnostic barriers and clinical biases, unequal treatment and coercive care, and outcomes.

Intersectional analyses in 3.9% revealed critical disparities in which transgender youth accounted for 14 % of emergency visits. Black bisexual men faced elevated risks of depression and cannabis use disorder. Institutional neglect affecting postpartum women and indigent adults brings together unemployment and structural racism, resulting in underdiagnosis of suicidality and lower access to essential contemporary pharmacological treatments in 5.5% of the results.

In 32.3% of the studies, involuntary hospital seclusion and the confinement of Black youth in psychiatric units are practices that evoke Fanonian punitive control over the body viewed as a constant external threat (96). Demographic factors such as Black race predict higher rates of clinical seclusion, revealing profound disparities in the management of aggressiveness and emotional containment within psychiatric inpatient settings (97).

In 34.6% of sources, delayed help-seeking, emergency service contact, police involvement, and compulsory admission appeared within the same care trajectories among Black patients. These co-occurring patterns warrant antiracist community-based responses, although their temporal ordering and causal relationships cannot be established through this descriptive evidence synthesis alone.

### 4.5 Epistemic hegemony and the decolonization of psychiatric knowledge

Because 89.0% of included sources originated in the United States, the synthesis largely reflects United States racial classifications, services, policies, and diagnostic practices. Consequently, findings may not transfer directly to African, Caribbean, Latin American, European, or other contexts where racialization and mental healthcare systems differ substantially.

The Jamaican construct known as shakatani emerges as a response of cultural resistance to the imposition of personality diagnoses that fail to capture the existential tensions of colonial oppression (98). Therefore, it is essential to question the universal validity of traditional psychiatric categories when applied to populations that have historically been the objects, rather than the subjects, of the construction of clinical medical knowledge (35).

Spirituality and religious well-being emerge as fundamental coping resources for Black patients experiencing a first episode of psychosis (99) in settings marked by profound material deprivation (100). There is an inverse correlation between symptom severity and existential well-being, suggesting that treatment should transcend pharmacology to reintegrate the subjectivity fragmented by racism (101).

Recovering Frantz Fanon’s thought is vital to decolonizing contemporary psychiatry and building a practice that truly recognizes and heals the denied humanity of the Black Brazilian subject (25). Only through a politically situated practice will it be possible to confront the roots of illness and promote mental health as a synonym for collective liberation and global racial justice (102).

### 4.6 Strengths and limitations

This review was strengthened by a preregistered protocol, broad database and gray-literature searches, unrestricted language and publication dates, and librarian-supported strategy development process. Independent screening and extraction, reciprocal coding verification, and transparent presentation of source frequencies further supported methodological consistency across a large, heterogeneous evidence base.

The evidence was geographically concentrated in the United States and varied in racial classification, population characteristics, clinical settings, study designs, and diagnostic practices. These features limit transferability, prevent direct equivalence across Black populations, and preclude conclusions about prevalence, effectiveness, causality, or certainty beyond the mapped sources.

Bibliographic searches, writing refinement, and reference indexing was not conducted, so sources contributed without weighting for methodological rigor, and findings cannot distinguish more robust evidence from weaker reports. Requiring clinician-established DSM or ICD diagnoses improved comparability but may have excluded instrument-based evidence and underrepresented settings with limited access to formal assessment.

## 5 Conclusion

This scoping review mapped heterogeneous evidence concerning mental disorders among Black populations, including diagnoses, associated factors, healthcare access, interventions, barriers, and psychosocial consequences. Patterns across sources linked psychiatric care with racialization, socioeconomic conditions, trauma, and service access, although their relative contributions could not be reliably determined.

The evidence was concentrated geographically and clinically, while racial information, non-United States contexts, community settings, and evaluations of interventions remained comparatively limited overall. These gaps constrain transferability and indicate priorities for research incorporating context-specific racial classifications, broader populations, diverse services, and transparent diagnostic procedures across regions.

Accordingly, findings support further investigation of culturally responsive, antiracist, and equity-oriented approaches, rather than directly establishing effectiveness of specific policies, practices, or interventions. Future studies should strengthen representation, document racial classification and diagnostic processes explicitly, and evaluate interventions before broader recommendations for mental healthcare are adopted.

## Data Availability

The original contributions presented in the study are included in the article/Supplementary material, further inquiries can be directed to the corresponding author.
