# Supplementary material for: Mental disorders in the Black population: a scoping review
Source: Front Public Health. 2026 Sep 3;14:1903601. doi: 10.3389/fpubh.2026.1903601 (PMC13581542; doi:10.3389/fpubh.2026.1903601)
Supplement: Supplementary file 1 [file Table_1.DOCX]

***Supplementary Material***

# 1 Appendix A. PRISMA 2020 Checklist

| **Section and Topic** | **Item #** | **Checklist item** | **Location where item is reported / status** |
| --- | --- | --- | --- |
| **TITLE** |  |  |  |
| Title | 1 | Identify the report as a systematic review. | Manuscript title: “Mental Disorders in the Black Population: A Scoping Review.” It identifies the type of review as a scoping review. |
| **ABSTRACT** |  |  |  |
| Abstract | 2 | See the PRISMA 2020 for Abstracts checklist. | Structured abstract: Objective, Introduction, Inclusion criteria, Methods, OSF Protocol, and Keywords. It is also recommended to consult PRISMA-ScR for abstracts, as this is a systematic review. |
| **INTRODUCTION** |  |  |  |
| Rationale | 3 | Describe the rationale for the review in the context of existing knowledge. | Introduction: paragraphs on the definition and global burden of TM, the Black population, racism, gaps in the literature, and the lack of published reviews on TM in the Black population. |
| Objectives | 4 | Provide an explicit statement of the objective(s) or question(s) the review addresses. | Resumo: Objective; Introdução: objetivo final; seção “Review question(s)”. Pergunta: “Quais são as evidências disponíveis sobre a ocorrência de TMs na PN ao redor do mundo?” |
| **METHODS** |  |  |  |
| Eligibility criteria | 5 | Specify the inclusion and exclusion criteria for the review and how studies were grouped for the syntheses. | Methods > Inclusion criteria: Participants, Concept, Context, Types of sources. Also described in Appendix II: Inclusion/Exclusion Criteria. |
| Information sources | 6 | Specify all databases, registers, websites, organisations, reference lists and other sources searched or consulted to identify studies. Specify the date when each source was last searched or consulted. | Methods > Information sources; Appendix A - Search strategy. Sources: MEDLINE/PubMed, CINAHL/EBSCO, APA PsycINFO, Scopus, Embase, PubMed PMC, Web of Science, BVS, BDTD, and ProQuest Theses; dates recorded on 04/02/2025, 04/03/2025, and updated on 02/27/2026. |
| Search strategy | 7 | Present the full search strategies for all databases, registers and websites, including any filters and limits used. | Methods > Search strategy; Appendix A - Search strategy. Complete strategies by database, with terms for adults, Black population, patients, mental disorders, diagnosis, DSM/ICD/scales; no language or date restrictions. |
| Selection process | 8 | Specify the methods used to decide whether a study met the inclusion criteria of the review, including how many reviewers screened each record and each report retrieved, whether they worked independently, and if applicable, details of automation tools used in the process. | Methods > Study/Source of evidence selection. Zotero and Rayyan; removal of duplicates; independent screening by RSS and YSR; discrepancies resolved by consensus or third-party reviewer PLFSR; PRISMA-ScR diagram in the Appendix. |
| Data collection process | 9 | Specify the methods used to collect data from reports, including how many reviewers collected data from each report, whether they worked independently, any processes for obtaining or confirming data from study investigators, and if applicable, details of automation tools used in the process. | Methods > Data extraction; Appendix II – Data extraction instrument. Data extracted using RSS and YSR with a custom tool; initial pilot study of 10% to refine the instrument. |
| Data items | 10a | List and define all outcomes for which data were sought. Specify whether all results that were compatible with each outcome domain in each study were sought, and if not, the methods used to decide which results to collect. | Methods > Data extraction; Appendix II. Data retrieved: participants/age group/gender/racial criteria; concept/type of TM/diagnostic categories/associated factors; context/country/health system/social markers; methodological design and main findings. |
| Data items | 10b | List and define all other variables for which data were sought. Describe any assumptions made about any missing or unclear information. | Appendix II - Data extraction instrument. Additional variables: citation details, country/state/municipality, context, participants, racial classification, disorders investigated, DSM/ICD/instruments, associated factors/barriers, interventions, policies, gaps, and methodological observations. Missing or ambiguous information should be coded as “not reported.” |
| Study risk of bias assessment | 11 | Specify the methods used to assess risk of bias in the included studies, including details of the tool(s) used, how many reviewers assessed each study and whether they worked independently, and if applicable, details of automation tools used in the process. | Not applicable/not required. JBI systematic reviews generally do not require a formal risk of bias assessment; the manuscript does not report a risk of bias tool. |
| Effect measures | 12 | Specify for each outcome the effect measure(s) used in the synthesis or presentation of results. | Not applicable. The review maps evidence and does not estimate comparative effect sizes; results are presented through narrative synthesis, tables, graphs, and descriptive statistics. |
| Synthesis methods | 13a | Describe the processes used to decide which studies were eligible for each synthesis. | Methods > Data analysis and presentation; Results. Eligibility for syntheses defined by PCC criteria and the fields of the data extraction tool; included studies were organized into tables and thematic/descriptive categories. |
| Synthesis methods | 13b | Describe any methods required to prepare the data for presentation or synthesis, such as handling of missing summary statistics, or data conversions. | Methods > Data analysis and presentation. Data prepared using standardized data extraction, thematic coding, descriptive frequencies, and treatment of missing fields as “not reported” where applicable. |
| Synthesis methods | 13c | Describe any methods used to tabulate or visually display results of individual studies and syntheses. | Methods > Data analysis and presentation; Results; Table 1; figures/tables from the synthesis. Results presented using tables, graphs, frequencies, and a narrative summary. |
| Synthesis methods | 13d | Describe any methods used to synthesize results and provide a rationale for the choice(s). If meta-analysis was performed, describe the model(s), method(s) to identify heterogeneity, and software used. | Methods > Data analysis and presentation. Synthesis by thematic analysis according to Braun and Clarke, a predominantly inductive approach, with systematic coding and iterative refinement of themes. No meta-analysis performed. |
| Synthesis methods | 13e | Describe any methods used to explore possible causes of heterogeneity among study results. | Not applicable. No subgroup analyses, meta-regression, or statistical exploration of heterogeneity were described; heterogeneity is addressed narratively in the discussion. |
| Synthesis methods | 13f | Describe any sensitivity analyses conducted to assess robustness of the synthesized results. | Not applicable. No sensitivity analyses were conducted. |
| Reporting bias assessment | 14 | Describe any methods used to assess risk of bias due to missing results in a synthesis. | Not applicable/not performed. There was no formal assessment of reporting bias or missing-out bias, consistent with a descriptive review. |
| Certainty assessment | 15 | Describe any methods used to assess certainty (or confidence) in the body of evidence for an outcome. | Not applicable/not performed. No formal assessment of the certainty of the evidence, such as GRADE or CERQual, was reported. |
| **RESULTS** |  |  |  |
| Study selection | 16a | Describe the results of the search and selection process, from the number of records identified in the search to the number of studies included in the review, ideally using a flow diagram. | Results > Study selection; PRISMA-ScR diagram in the Appendix. Project data: the search identified 2,131 records; 235 included after screening; the update added 16 documents; 254/261 available for final data extraction; 7/261 not located. |
| Study selection | 16b | Cite studies that might appear to meet the inclusion criteria, but which were excluded, and explain why they were excluded. | Results > Study selection / PRISMA-ScR Appendix. It is recommended to include a list or note of the full-text articles excluded, with reasons if available; the available sources only state that 7/261 included references could not be located for data extraction. |
| Study characteristics | 17 | Cite each included study and present its characteristics. | Results; Table 1; Data Extraction Tool. Extracted characteristics: author/year/title/journal, country, context, participants, study design, diagnostic classification, disorders, associated factors, interventions, and findings. |
| Risk of bias in studies | 18 | Present assessments of risk of bias for each included study. | Not applicable/not performed. Risk of bias assessment was not planned or reported. |
| Results of individual studies | 19 | For all outcomes, present, for each study: summary statistics for each group and effect estimate with precision, ideally using structured tables or plots. | Results; Table 1; Data Extraction Tool. As this is a scoping review, there are no individual effect estimates; the studies are presented by characteristics and findings extracted. |
| Results of syntheses | 20a | For each synthesis, briefly summarise the characteristics and risk of bias among contributing studies. | Results; Discussion. Descriptive syntheses by study characteristics and thematic categories; no summary by risk of bias. |
| Results of syntheses | 20b | Present results of all statistical syntheses conducted. | Not applicable. No meta-analysis or inferential statistical synthesis was conducted. |
| Results of syntheses | 20c | Present results of all investigations of possible causes of heterogeneity among study results. | Not applicable. No statistical investigation of heterogeneity was conducted. |
| Results of syntheses | 20d | Present results of all sensitivity analyses conducted to assess the robustness of the synthesized results. | Not applicable. Sensitivity analyses were not conducted. |
| Reporting biases | 21 | Present assessments of risk of bias due to missing results for each synthesis assessed. | Not applicable/not performed. Assessment of reporting bias was not presented. |
| Certainty of evidence | 22 | Present assessments of certainty (or confidence) in the body of evidence for each outcome assessed. | Not applicable/not performed. Formal assessment of the certainty/confidence of the evidence was not presented. |
| **DISCUSSION** |  |  |  |
| Discussion | 23a | Provide a general interpretation of the results in the context of other evidence. | Discussion. General interpretation of the findings in light of structural racism, psychiatric diagnosis, care, inequities, the geography of scientific production, and a Fanonian reading of clinical practice. |
| Discussion | 23b | Discuss any limitations of the evidence included in the review. | Discussion > limitations of the evidence. Limitations: geographic concentration, predominance of U.S. data, heterogeneity in racial/diagnostic classification, gaps in low- and middle-income countries, and incomplete racial data. |
| Discussion | 23c | Discuss any limitations of the review processes used. | Discussion > limitations of the review process. Potential limitations: reliance on selected databases, availability of full-text articles, 7/261 references not located, data extraction possible in 254/261, and heterogeneity of extracted data. |
| Discussion | 23d | Discuss implications of the results for practice, policy, and future research. | Discussion > Implications. Implications for anti-racist clinical practice, public policy, qualification of racial information, development of culturally situated instruments, and future research agenda. |
| **OTHER INFORMATION** |  |  |  |
| Registration and protocol | 24a | Provide registration information for the review, including register name and registration number, or state that the review was not registered. | Abstract: Protocol OSF DOI 10.17605/OSF.IO/WQAVD; Methods: protocol registered on the Open Science Framework. |
| Registration and protocol | 24b | Indicate where the review protocol can be accessed, or state that a protocol was not prepared. | Abstract and Methods: protocol available on OSF via DOI 10.17605/OSF.IO/WQAVD. |
| Registration and protocol | 24c | Describe and explain any amendments to information provided at registration or in the protocol. | Not reported in the available sources. If there were amendments to the protocol, include a description and justification; otherwise, state that there were no amendments. |
| Support | 25 | Describe sources of financial or non-financial support for the review, and the role of the funders or sponsors in the review. | Not reported in the available sources. Include sources of financial/non-financial support and the role of funders, or state that there was no funding. |
| Competing interests | 26 | Declare any competing interests of review authors. | Not reported in the available sources. Include an explicit statement of the authors’ conflicts of interest. |
| Availability of data, code and other materials | 27 | Report which materials are publicly available and where they can be found. | Other information / Data availability statement to be inserted. Available sources: Appendix A (strategy), Appendix II (extraction tool), “Excel Data Extraction Tool” and “New Synthesis of Mental Disorders” spreadsheets. Indicate whether they will be made available in the OSF, as a supplement, or upon request. |

Page MJ, McKenzie JE, Bossuyt PM, Boutron I, Hoffmann TC, Mulrow CD, et al. The PRISMA 2020 statement: an updated guideline for reporting systematic reviews. BMJ. 2021;372:n71. doi: 10.1136/bmj.n71. Licença CC BY 4.0.
