# Supplementary material for: Mental disorders in the Black population: a scoping review
Source: Front Public Health. 2026 Sep 3;14:1903601. doi: 10.3389/fpubh.2026.1903601 (PMC13581542; doi:10.3389/fpubh.2026.1903601)
Supplement: Supplementary file 2 [file Table_2.DOCX]

***Supplementary Material***

# 1 Appendix B. Search strategy

**Search strategy - 02/04/2025**

| **Source** | **Strategy** | **Nº of Articles and Date** | **Date** |
| --- | --- | --- | --- |
| **PUBMED** | (Adult OR Adults OR "Young Adult" OR "Young Adults" OR "Middle Aged" OR "Middle Age" OR Aged OR Elderly OR "Older Adults" OR "Older people" OR "Older person" OR Senior OR "Oldest adults" OR Elders OR "Very elderly" OR "Aged, 80 and over" OR "Oldest Old" OR Centenarians OR Centenarian OR Nonagenarians OR Nonagenarian OR Octogenarians OR Octogenarian) AND (((((((((Black People[MeSH Terms]) OR ("Black People"[Title/Abstract] OR "Black Peoples"[Title/Abstract] OR "People, Black"[Title/Abstract] OR "Black Person"[Title/Abstract] OR "Black Persons"[Title/Abstract] OR "Negroid Race"[Title/Abstract] OR "Negroid Races"[Title/Abstract] OR "Race, Negroid"[Title/Abstract] OR "African Continental Ancestry Group"[Title/Abstract])) OR ("Black Population"[Title/Abstract] OR "Black man"[Title/Abstract] OR "Black race"[Title/Abstract] OR Negroid[Title/Abstract] OR "Negroid race"[Title/Abstract] OR Negroids[Title/Abstract])) OR ((Black or African American[MeSH Terms]) OR ("Black or African American"[Title/Abstract] OR "Black Americans"[Title/Abstract] OR "American, Black"[Title/Abstract] OR "Black American"[Title/Abstract] OR Blacks[Title/Abstract] OR Negroes[Title/Abstract] OR Negro[Title/Abstract] OR "African Americans"[Title/Abstract] OR "African American"[Title/Abstract] OR "American, African"[Title/Abstract] OR "Afro-American"[Title/Abstract] OR "Afro American"[Title/Abstract] OR "Afro-Americans"[Title/Abstract] OR "Afro Americans"[Title/Abstract] OR "African-Americans"[Title/Abstract] OR "African-American"[Title/Abstract]))) OR ("American blacks"[Title/Abstract] OR "American Negro"[Title/Abstract] OR "black American"[Title/Abstract] OR "black or African American"[Title/Abstract])) AND ((Patients[MeSH Terms]) OR (Patients[Title/Abstract] OR Patient[Title/Abstract] OR Clients[Title/Abstract] OR Client[Title/Abstract]))) AND ((((Mental Disorders[MeSH Terms]) OR ("Mental Disorders"[Title/Abstract] OR "Mental Disorder"[Title/Abstract] OR "Psychiatric Disorders"[Title/Abstract] OR "Psychiatric Disorder"[Title/Abstract] OR "Psychiatric Diseases"[Title/Abstract] OR "Psychiatric Disease"[Title/Abstract] OR "Psychiatric Illness"[Title/Abstract] OR "Psychiatric Illnesses"[Title/Abstract] OR "Psychiatric Illness"[Title/Abstract] OR "Mental Illness"[Title/Abstract] OR "Mental Illnesses"[Title/Abstract] OR "Behavior Disorders"[Title/Abstract] OR "Psychiatric Diagnosis"[Title/Abstract] OR "Severe Mental Disorder"[Title/Abstract] OR "Severe Mental Disorders"[Title/Abstract])) OR ("mental disease"[Title/Abstract])) AND (((Diagnosis[MeSH Terms]) OR (Diagnosis[Title/Abstract] OR Diagnoses[Title/Abstract] OR Diagnose[Title/Abstract] OR "Diagnoses and Examinations"[Title/Abstract] OR "Diagnoses and Examination"[Title/Abstract] OR "Examination and Diagnoses"[Title/Abstract] OR "Examinations and Diagnoses"[Title/Abstract])) OR ("diagnostic screening"[Title/Abstract] OR diagnostics[Title/Abstract] OR "disease diagnosis"[Title/Abstract]))))) AND ((((((Diagnostic and Statistical Manual of Mental Disorders[MeSH Terms]) OR ("Diagnostic and Statistical Manual of Mental Disorders"[Title/Abstract] OR DSM-IV[Title/Abstract] OR DSM-II[Title/Abstract] OR DSM-III[Title/Abstract] OR DSM-V[Title/Abstract])) OR ("DSM classification"[Title/Abstract])) OR ((International Classification of Diseases[MeSH Terms]) OR ("International Classification of Diseases"[Title/Abstract] OR "International Classification of Disease"[Title/Abstract] OR "Disease International Classification"[Title/Abstract] OR "International Statistical Classification of Diseases and Related Health Problems"[Title/Abstract] OR "ICD Codes"[Title/Abstract] OR "ICD Code"[Title/Abstract] OR "International Classification of Disease Codes"[Title/Abstract] OR ICD-10[Title/Abstract] OR ICD-9[Title/Abstract] OR ICD-9-CM[Title/Abstract] OR ICD-10-CM[Title/Abstract] OR ICD-11[Title/Abstract] OR "ICD-10 Procedure Coding System"[Title/Abstract] OR "ICD 10 Procedure Coding System"[Title/Abstract] OR ICD-10-PCS[Title/Abstract]))) OR ((Psychiatric Status Rating Scales[MeSH Terms]) OR ("Psychiatric Status Rating Scales"[Title/Abstract] OR "Katz Adjustment Scales"[Title/Abstract] OR "Wittenborn Scales"[Title/Abstract] OR "Edinburgh Postnatal Depression Scale"[Title/Abstract] OR "Mini International Neuropsychiatric Interview"[Title/Abstract]))) OR ("psychiatric rating scale"[Title/Abstract]))) | **02/04/2025**  **455** | **27/02/2026**  **475** |
| **PUBMED PMC** | ((((((Adult OR Adults OR "Young Adult" OR "Young Adults" OR "Middle Aged" OR "Middle Age" OR Aged OR Elderly OR "Older Adults" OR "Older people" OR "Older person" OR Senior OR "Oldest adults" OR Elders OR "Very elderly" OR "Aged, 80 and over" OR "Oldest Old" OR Centenarians OR Centenarian OR Nonagenarians OR Nonagenarian OR Octogenarians OR Octogenarian)) AND ((((((Black People[MeSH Terms]) OR ("Black People"[Title/Abstract] OR "Black Peoples"[Title/Abstract] OR "People, Black"[Title/Abstract] OR "Black Person"[Title/Abstract] OR "Black Persons"[Title/Abstract] OR "Negroid Race"[Title/Abstract] OR "Negroid Races"[Title/Abstract] OR "Race, Negroid"[Title/Abstract] OR "African Continental Ancestry Group"[Title/Abstract])) OR ("Black Population"[Title/Abstract] OR "Black man"[Title/Abstract] OR "Black race"[Title/Abstract] OR Negroid[Title/Abstract] OR "Negroid race"[Title/Abstract] OR Negroids[Title/Abstract])) OR (Black or African American[MeSH Terms])) OR ("Black or African American"[Title/Abstract] OR "Black Americans"[Title/Abstract] OR "American, Black"[Title/Abstract] OR "Black American"[Title/Abstract] OR Blacks[Title/Abstract] OR Negroes[Title/Abstract] OR Negro[Title/Abstract] OR "African Americans"[Title/Abstract] OR "African American"[Title/Abstract] OR "American, African"[Title/Abstract] OR "Afro-American"[Title/Abstract] OR "Afro American"[Title/Abstract] OR "Afro-Americans"[Title/Abstract] OR "Afro Americans"[Title/Abstract] OR "African-Americans"[Title/Abstract] OR "African-American"[Title/Abstract])) OR ("American blacks"[Title/Abstract] OR "American Negro"[Title/Abstract] OR "black American"[Title/Abstract] OR "black or African American"[Title/Abstract]))) AND ((Patients[MeSH Terms]) OR (Patients[Title/Abstract] OR Patient[Title/Abstract] OR Clients[Title/Abstract] OR Client[Title/Abstract]))) AND (((Mental Disorders[MeSH Terms]) OR ("Mental Disorders"[Title/Abstract] OR "Mental Disorder"[Title/Abstract] OR "Psychiatric Disorders"[Title/Abstract] OR "Psychiatric Disorder"[Title/Abstract] OR "Psychiatric Diseases"[Title/Abstract] OR "Psychiatric Disease"[Title/Abstract] OR "Psychiatric Illnesses"[Title/Abstract] OR "Psychiatric Illness"[Title/Abstract] OR "Mental Illness"[Title/Abstract] OR "Mental Illnesses"[Title/Abstract] OR "Behavior Disorders"[Title/Abstract] OR "Psychiatric Diagnosis"[Title/Abstract] OR "Severe Mental Disorder"[Title/Abstract] OR "Severe Mental Disorders"[Title/Abstract])) OR ("mental disease"[Title/Abstract]))) AND (((Diagnosis[MeSH Terms]) OR (Diagnosis[Title/Abstract] OR Diagnoses[Title/Abstract] OR Diagnose[Title/Abstract] OR "Diagnoses and Examinations"[Title/Abstract] OR "Diagnoses and Examination"[Title/Abstract] OR "Examination and Diagnoses"[Title/Abstract] OR "Examinations and Diagnoses"[Title/Abstract])) OR ("diagnostic screening"[Title/Abstract] OR diagnostics[Title/Abstract] OR "disease diagnosis"[Title/Abstract]))) AND (((((((((Diagnostic and Statistical Manual of Mental Disorders[MeSH Terms]) OR ("Diagnostic and Statistical Manual of Mental Disorders"[Title/Abstract] OR DSM-IV[Title/Abstract] OR DSM-II[Title/Abstract] OR DSM-III[Title/Abstract] OR DSM-V[Title/Abstract])) OR ("DSM classification"[Title/Abstract])) OR (International Classification of Diseases[MeSH Terms])) OR ("International Classification of Diseases"[Title/Abstract] OR "International Classification of Disease"[Title/Abstract] OR "Disease International Classification"[Title/Abstract] OR "International Statistical Classification of Diseases and Related Health Problems"[Title/Abstract] OR "ICD Codes"[Title/Abstract] OR "ICD Code"[Title/Abstract] OR "International Classification of Disease Codes"[Title/Abstract] OR ICD-10[Title/Abstract] OR ICD-9[Title/Abstract] OR ICD-9-CM[Title/Abstract] OR ICD-10-CM[Title/Abstract] OR ICD-11[Title/Abstract] OR "ICD-10 Procedure Coding System"[Title/Abstract] OR "ICD 10 Procedure Coding System"[Title/Abstract] OR ICD-10-PCS[Title/Abstract])) OR ("International Classification of Diseases 10th Revision"[Title/Abstract] OR "International Classification of Diseases Tenth Revision"[Title/Abstract])) OR (Psychiatric Status Rating Scales[MeSH Terms])) OR ("Psychiatric Status Rating Scales"[Title/Abstract] OR "Katz Adjustment Scales"[Title/Abstract] OR "Wittenborn Scales"[Title/Abstract] OR "Edinburgh Postnatal Depression Scale"[Title/Abstract] OR "Mini International Neuropsychiatric Interview"[Title/Abstract])) OR ("psychiatric rating scale"[Title/Abstract])) | **02/04/2025**  **122** | **27/02/2026**  **122** |
| **BDTD** | (Todos os campos:"Manual Diagnóstico e Estatístico de Transtornos Mentais" OR "Classificação Internacional de Doenças" OR "Escalas de Graduação Psiquiátrica" OR ICD-10 E Todos os campos:("População Negra" OR Negro OR "Afro-Americano") AND (Paciente OR Pacientes) E Todos os campos:("Transtornos Mentais" OR "Transtorno MentaL") AND Diagnóstico E Todos os campos:Adulto OR "Adulto Jovem" OR "Pessoa de Meia-Idade" OR Idoso ) | **03/04/2025**  **0** | **27/02/2026**  **0** |
| **BVS / BIREME**  **MEDLINE**  **LILACS** | ((adult OR adults OR "Young Adult" OR "Young Adults" OR "Middle Aged" OR "Middle Age" OR aged OR elderly OR "Older Adults" OR "Older people" OR "Older person" OR senior OR "Oldest adults" OR elders OR "Very elderly" OR "Aged, 80 and over" OR "Oldest Old" OR centenarians OR centenarian OR nonagenarians OR nonagenarian OR octogenarians OR octogenarian)) AND (("Black People" OR "Black Peoples" OR "People, Black" OR "Black Person" OR "Black Persons" OR "Negroid Race" OR "Negroid Races" OR "Race, Negroid" OR "African Continental Ancestry Group") OR ("Black Population" OR "Black man" OR "Black race" OR negroid OR "Negroid race" OR negroids ) OR ("Black or African American" OR "Black Americans" OR "American, Black" OR "Black American" OR blacks OR negroes OR negro OR "African Americans" OR "African American" OR "American, African" OR "Afro-American" OR "Afro American" OR "Afro-Americans" OR "Afro Americans" OR "African-Americans" OR "African-American") OR ("American blacks" OR "American Negro" OR "black American" OR "black or African American")) AND ((patients OR patient OR clients OR client)) AND (("Mental Disorders" OR "Mental Disorder" OR "Psychiatric Disorders" OR "Psychiatric Disorder" OR "Psychiatric Diseases" OR "Psychiatric Disease" OR "Psychiatric Illnesses" OR "Psychiatric Illness" OR "Mental Illness" OR "Mental Illnesses" OR "Behavior Disorders" OR "Psychiatric Diagnosis" OR "Severe Mental Disorder" OR "Severe Mental Disorders") OR ("mental disease")) AND ((diagnosis OR diagnoses OR diagnose OR "Diagnoses and Examinations" OR "Diagnoses and Examination" OR "Examination and Diagnoses" OR "Examinations and Diagnoses") OR ("diagnostic screening" OR diagnostics OR "disease diagnosis")) AND (("Diagnostic and Statistical Manual of Mental Disorders" OR dsm-iv OR dsm-ii OR dsm-iii OR dsm-v) OR ("DSM classification" ) OR ("International Classification of Diseases" OR "International Classification of Disease" OR "Disease International Classification" OR "International Statistical Classification of Diseases and Related Health Problems" OR "ICD Codes" OR "ICD Code" OR "International Classification of Disease Codes" OR icd-10 OR icd-9 OR icd-9-cm OR icd-10-cm OR icd-11 OR "ICD-10 Procedure Coding System" OR "ICD 10 Procedure Coding System" OR icd-10-pcs) OR ("International Classification of Diseases 10th Revision" OR "International Classification of Diseases Tenth Revision") OR ("Psychiatric Status Rating Scales" OR "Katz Adjustment Scales" OR "Wittenborn Scales" OR "Edinburgh Postnatal Depression Scale" OR "Mini International Neuropsychiatric Interview") OR ("psychiatric rating scale" )) AND instance:"regional" | **03/04/2025**  **156** | **27/02/2026**  **164** |
| **EBSCOHOST** | (Adult OR Adults OR "Young Adult" OR "Young Adults" OR "Middle Aged" OR "Middle Age" OR Aged OR Elderly OR "Older Adults" OR "Older people" OR "Older person" OR Senior OR "Oldest adults" OR Elders OR "Very elderly" OR "Aged, 80 and over" OR "Oldest Old" OR Centenarians OR Centenarian OR Nonagenarians OR Nonagenarian OR Octogenarians OR Octogenarian) AND ((MM "Black People+" OR TI ("Black People" OR "Black Peoples" OR "People, Black" OR "Black Person" OR "Black Persons" OR "Negroid Race" OR "Negroid Races" OR "Race, Negroid" OR "African Continental Ancestry Group") OR AB ("Black People" OR "Black Peoples" OR "People, Black" OR "Black Person" OR "Black Persons" OR "Negroid Race" OR "Negroid Races" OR "Race, Negroid" OR "African Continental Ancestry Group") OR TI ("Black Population" OR "Black man" OR "Black race" OR Negroid OR "Negroid race" OR Negroids) OR AB ("Black Population" OR "Black man" OR "Black race" OR Negroid OR "Negroid race" OR Negroids)) OR ((MM "Black or African American") OR TI ("Black or African American" OR "Black Americans" OR "American, Black" OR "Black American" OR Blacks OR Negroes OR Negro OR "African Americans" OR "African American" OR "American, African" OR "Afro-American" OR "Afro American" OR "Afro-Americans" OR "Afro Americans" OR "African-Americans" OR "African-American") OR AB ("Black or African American" OR "Black Americans" OR "American, Black" OR "Black American" OR Blacks OR Negroes OR Negro OR "African Americans" OR "African American" OR "American, African" OR "Afro-American" OR "Afro American" OR "Afro-Americans" OR "Afro Americans" OR "African-Americans" OR "African-American") OR TI ("American blacks" OR "American Negro" OR "black American" OR "black or African American") OR AB ("American blacks" OR "American Negro" OR "black American" OR "black or African American"))) AND (MM "Patients+" OR TI (Patients OR Patient OR Clients OR Client) OR AB (Patients OR Patient OR Clients OR Client)) AND (MM "Mental Disorders+" OR TI ("Mental Disorders" OR "Mental Disorder" OR "Psychiatric Disorders" OR "Psychiatric Disorder" OR "Psychiatric Diseases" OR "Psychiatric Disease" OR "Psychiatric Illnesses" OR "Psychiatric Illness" OR "Mental Illness" OR "Mental Illnesses" OR "Behavior Disorders" OR "Psychiatric Diagnosis" OR "Severe Mental Disorder" OR "Severe Mental Disorders") OR AB ("Mental Disorders" OR "Mental Disorder" OR "Psychiatric Disorders" OR "Psychiatric Disorder" OR "Psychiatric Diseases" OR "Psychiatric Disease" OR "Psychiatric Illnesses" OR "Psychiatric Illness" OR "Mental Illness" OR "Mental Illnesses" OR "Behavior Disorders" OR "Psychiatric Diagnosis" OR "Severe Mental Disorder" OR "Severe Mental Disorders") OR TI "mental disease" OR AB "mental disease") AND (MM "Diagnosis+" OR TI (Diagnosis OR Diagnoses OR Diagnose OR "Diagnoses and Examinations" OR "Diagnoses and Examination" OR "Examination and Diagnoses" OR "Examinations and Diagnoses") OR AB (Diagnosis OR Diagnoses OR Diagnose OR "Diagnoses and Examinations" OR "Diagnoses and Examination" OR "Examination and Diagnoses" OR "Examinations and Diagnoses") OR TI ("diagnostic screening" OR diagnostics OR "disease diagnosis") OR AB ("diagnostic screening" OR diagnostics OR "disease diagnosis")) AND (((MM "Diagnostic and Statistical Manual of Mental Disorders") OR TI ("Diagnostic and Statistical Manual of Mental Disorders" OR DSM-IV OR DSM-II OR DSM-III OR DSM-V) OR AB ("Diagnostic and Statistical Manual of Mental Disorders" OR DSM-IV OR DSM-II OR DSM-III OR DSM-V) OR TI "DSM classification" OR AB "DSM classification") OR (MM "International Classification of Diseases" OR TI ("International Classification of Diseases" OR "International Classification of Disease" OR "Disease International Classification" OR "International Statistical Classification of Diseases and Related Health Problems" OR "ICD Codes" OR "ICD Code" OR "International Classification of Disease Codes" OR ICD-10 OR ICD-9 OR ICD-9-CM OR ICD-10-CM OR ICD-11 OR "ICD-10 Procedure Coding System" OR "ICD 10 Procedure Coding System" OR ICD-10-PCS) OR AB ("International Classification of Diseases" OR "International Classification of Disease" OR "Disease International Classification" OR "International Statistical Classification of Diseases and Related Health Problems" OR "ICD Codes" OR "ICD Code" OR "International Classification of Disease Codes" OR ICD-10 OR ICD-9 OR ICD-9-CM OR ICD-10-CM OR ICD-11 OR "ICD-10 Procedure Coding System" OR "ICD 10 Procedure Coding System" OR ICD-10-PCS) OR TI ("International Classification of Diseases 10th Revision" OR "International Classification of Diseases Tenth Revision") OR AB ("International Classification of Diseases 10th Revision" OR "International Classification of Diseases Tenth Revision")) OR (MM "Psychiatric Status Rating Scales+" OR TI ("Psychiatric Status Rating Scales" OR "Katz Adjustment Scales" OR "Wittenborn Scales" OR "Edinburgh Postnatal Depression Scale" OR "Mini International Neuropsychiatric Interview") OR AB ("Psychiatric Status Rating Scales" OR "Katz Adjustment Scales" OR "Wittenborn Scales" OR "Edinburgh Postnatal Depression Scale" OR "Mini International Neuropsychiatric Interview") OR TI "psychiatric rating scale" OR AB "psychiatric rating scale")) | **03/04/2025**  **101** | **27/02/2026**  **112** |
| **SCOPUS** | ( ALL ( adult OR adults OR "Young Adult" OR "Young Adults" OR "Middle Aged" OR "Middle Age" OR aged OR elderly OR "Older Adults" OR "Older people" OR "Older person" OR senior OR "Oldest adults" OR elders OR "Very elderly" OR "Aged, 80 and over" OR "Oldest Old" OR centenarians OR centenarian OR nonagenarians OR nonagenarian OR octogenarians OR octogenarian ) ) AND ( ( TITLE-ABS-KEY ( "Black People" OR "Black Peoples" OR "People, Black" OR "Black Person" OR "Black Persons" OR "Negroid Race" OR "Negroid Races" OR "Race, Negroid" OR "African Continental Ancestry Group" ) OR TITLE-ABS-KEY ( "Black Population" OR "Black man" OR "Black race" OR negroid OR "Negroid race" OR negroids ) OR TITLE-ABS-KEY ( "Black or African American" OR "Black Americans" OR "American, Black" OR "Black American" OR blacks OR negroes OR negro OR "African Americans" OR "African American" OR "American, African" OR "Afro-American" OR "Afro American" OR "Afro-Americans" OR "Afro Americans" OR "African-Americans" OR "African-American" ) OR TITLE-ABS-KEY ( "American blacks" OR "American Negro" OR "black American" OR "black or African American" ) ) ) AND ( TITLE-ABS-KEY ( patients OR patient OR clients OR client ) ) AND ( ( TITLE-ABS-KEY ( "Mental Disorders" OR "Mental Disorder" OR "Psychiatric Disorders" OR "Psychiatric Disorder" OR "Psychiatric Diseases" OR "Psychiatric Disease" OR "Psychiatric Illnesses" OR "Psychiatric Illness" OR "Mental Illness" OR "Mental Illnesses" OR "Behavior Disorders" OR "Psychiatric Diagnosis" OR "Severe Mental Disorder" OR "Severe Mental Disorders" ) OR TITLE-ABS-KEY ( "mental disease" ) ) ) AND ( ( TITLE-ABS-KEY ( diagnosis OR diagnoses OR diagnose OR "Diagnoses and Examinations" OR "Diagnoses and Examination" OR "Examination and Diagnoses" OR "Examinations and Diagnoses" ) OR TITLE-ABS-KEY ( "diagnostic screening" OR diagnostics OR "disease diagnosis" ) ) ) AND ( ( TITLE-ABS-KEY ( "Diagnostic and Statistical Manual of Mental Disorders" OR dsm-iv OR dsm-ii OR dsm-iii OR dsm-v ) OR TITLE-ABS-KEY ( "DSM classification" ) OR TITLE-ABS-KEY ( "International Classification of Diseases" OR "International Classification of Disease" OR "Disease International Classification" OR "International Statistical Classification of Diseases and Related Health Problems" OR "ICD Codes" OR "ICD Code" OR "International Classification of Disease Codes" OR icd-10 OR icd-9 OR icd-9-cm OR icd-10-cm OR icd-11 OR "ICD-10 Procedure Coding System" OR "ICD 10 Procedure Coding System" OR icd-10-pcs ) OR TITLE-ABS-KEY ( "International Classification of Diseases 10th Revision" OR "International Classification of Diseases Tenth Revision" ) OR TITLE-ABS-KEY ( "Psychiatric Status Rating Scales" OR "Katz Adjustment Scales" OR "Wittenborn Scales" OR "Edinburgh Postnatal Depression Scale" OR "Mini International Neuropsychiatric Interview" ) OR TITLE-ABS-KEY ( "psychiatric rating scale" ) ) ) | **02/04/2025**  **365** | **27/02/2026**  **388** |
| **WEB OF SCIENCE** | Adult OR Adults OR "Young Adult" OR "Young Adults" OR "Middle Aged" OR "Middle Age" OR Aged OR Elderly OR "Older Adults" OR "Older people" OR "Older person" OR Senior OR "Oldest adults" OR Elders OR "Very elderly" OR "Aged, 80 and over" OR "Oldest Old" OR Centenarians OR Centenarian OR Nonagenarians OR Nonagenarian OR Octogenarians OR Octogenarian AND "Black People" OR "Black Peoples" OR "People, Black" OR "Black Person" OR "Black Persons" OR "Negroid Race" OR "Negroid Races" OR "Race, Negroid" OR "African Continental Ancestry Group" (Topic) OR "Black Population" OR "Black man" OR "Black race" OR Negroid OR "Negroid race" OR Negroids (Topic) OR "Black or African American" OR "Black Americans" OR "American, Black" OR "Black American" OR Blacks OR Negroes OR Negro OR "African Americans" OR "African American" OR "American, African" OR "Afro-American" OR "Afro American" OR "Afro-Americans" OR "Afro Americans" OR "African-Americans" OR "African-American" (Topic) OR "American blacks" OR "American Negro" OR "black American" OR "black or African American" (Topic) and Preprint Citation Index (Exclude – Database) AND Patients OR Patient OR Clients OR Client AND "Mental Disorders" OR "Mental Disorder" OR "Psychiatric Disorders" OR "Psychiatric Disorder" OR "Psychiatric Diseases" OR "Psychiatric Disease" OR "Psychiatric Illnesses" OR "Psychiatric Illness" OR "Mental Illness" OR "Mental Illnesses" OR "Behavior Disorders" OR "Psychiatric Diagnosis" OR "Severe Mental Disorder" OR "Severe Mental Disorders" (Topic) OR "mental disease" (Topic) and Preprint Citation Index (Exclude – Database) AND Diagnosis OR Diagnoses OR Diagnose OR "Diagnoses and Examinations" OR "Diagnoses and Examination" OR "Examination and Diagnoses" OR "Examinations and Diagnoses" (Topic) OR "diagnostic screening" OR diagnostics OR "disease diagnosis" (Topic) and Preprint Citation Index (Exclude – Database) AND "Diagnostic and Statistical Manual of Mental Disorders" OR DSM-IV OR DSM-II OR DSM-III OR DSM-V (Topic) OR "DSM classification" (Topic) OR "International Classification of Diseases" OR "International Classification of Disease" OR "Disease International Classification" OR "International Statistical Classification of Diseases and Related Health Problems" OR "ICD Codes" OR "ICD Code" OR "International Classification of Disease Codes" OR ICD-10 OR ICD-9 OR ICD-9-CM OR ICD-10-CM OR ICD-11 OR "ICD-10 Procedure Coding System" OR "ICD 10 Procedure Coding System" OR ICD-10-PCS (Topic) OR "International Classification of Diseases 10th Revision" OR "International Classification of Diseases Tenth Revision" (Topic) OR "Psychiatric Status Rating Scales" OR "Katz Adjustment Scales" OR "Wittenborn Scales" OR "Edinburgh Postnatal Depression Scale" OR "Mini International Neuropsychiatric Interview" (Topic) OR "psychiatric rating scale" (Topic) and Preprint Citation Index (Exclude – Database)  [https://www.webofscience.com/wos/alldb/summary/81c2ede4-1894-4d39-9ee0-35ac2043dbac-0157a231a2/relevance/1](https://www.webofscience.com/wos/alldb/summary/81c2ede4-1894-4d39-9ee0-35ac2043dbac-0157a231a2/relevance/1%20) | **02/04/2025**  **1.242** | **27/02/2026**  **1.106** |
| **EMBASE** | (adult OR adults OR 'young adult' OR 'young adults' OR 'middle aged' OR 'middle age' OR aged OR elderly OR 'older adults' OR 'older people' OR 'older person' OR senior OR 'oldest adults' OR elders OR 'very elderly' OR 'aged, 80 and over' OR 'oldest old' OR centenarians OR centenarian OR nonagenarians OR nonagenarian OR octogenarians OR octogenarian) AND ('black person'/syn OR 'african american'/syn) AND 'patient'/syn AND 'mental disease'/syn AND 'diagnosis'/syn AND ('dsm-5'/syn OR (diagnostic:ti,ab,kw AND 'statistical manual of mental disorders':ti,ab,kw) OR 'dsm iv':ti,ab,kw OR 'dsm ii':ti,ab,kw OR 'dsm iii':ti,ab,kw OR 'dsm v':ti,ab,kw OR 'icd-10'/syn OR 'psychological rating scale'/syn) | **02/04/2025**  **443** | **27/02/2026**  **527** |
| **PSYCINFO** | ((Any Field: (Adult) OR Any Field: (Adults) OR Any Field: ("Young Adult") OR Any Field: ("Young Adults") OR Any Field: ("Middle Aged") OR Any Field: ("Middle Age") OR Any Field: (Aged) OR Any Field: (Elderly) OR Any Field: ("Older Adults") OR Any Field: ("Older people") OR Any Field: ("Older person") OR Any Field: (Senior) OR Any Field: ("Oldest adults") OR Any Field: (Elders) OR Any Field: ("Very elderly") OR Any Field: ("Aged, 80 and over") OR Any Field: ("Oldest Old") OR Any Field: (Centenarians) OR Any Field: (Centenarian) OR Any Field: (Nonagenarians) OR Any Field: (Nonagenarian) OR Any Field: (Octogenarians) OR Any Field: (Octogenarian))) AND ((title: ("Black People") OR title: ("Black Peoples") OR title: ("People, Black") OR title: ("Black Person") OR title: ("Black Persons") OR title: ("Negroid Race") OR title: ("Negroid Races") OR title: ("Race, Negroid") OR title: ("African Continental Ancestry Group")) OR (abstract: ("Black People") OR abstract: ("Black Peoples") OR abstract: ("People, Black") OR abstract: ("Black Person") OR abstract: ("Black Persons") OR abstract: ("Negroid Race") OR abstract: ("Negroid Races") OR abstract: ("Race, Negroid") OR abstract: ("African Continental Ancestry Group")) OR (title: ("Black Population") OR title: ("Black man") OR title: ("Black race") OR title: (Negroid) OR title: ("Negroid race") OR title: (Negroids)) OR (abstract: ("Black Population") OR abstract: ("Black man") OR abstract: ("Black race") OR abstract: (Negroid) OR abstract: ("Negroid race") OR abstract: (Negroids)) OR (title: ("Black or African American") OR title: ("Black Americans") OR title: ("American, Black") OR title: ("Black American") OR title: (Blacks) OR title: (Negroes) OR title: (Negro) OR title: ("African Americans") OR title: ("African American") OR title: ("American, African") OR title: ("Afro-American") OR title: ("Afro American") OR title: ("Afro-Americans") OR title: ("Afro Americans") OR title: ("African-Americans") OR title: ("African-American")) OR (abstract: ("Black or African American") OR abstract: ("Black Americans") OR abstract: ("American, Black") OR abstract: ("Black American") OR abstract: (Blacks) OR abstract: (Negroes) OR abstract: (Negro) OR abstract: ("African Americans") OR abstract: ("African American") OR abstract: ("American, African") OR abstract: ("Afro-American") OR abstract: ("Afro American") OR abstract: ("Afro-Americans") OR abstract: ("Afro Americans") OR abstract: ("African-Americans") OR abstract: ("African-American")) OR (title: ("American blacks") OR title: ("American Negro") OR title: ("black American") OR title: ("black or African American")) OR (abstract: ("American blacks") OR abstract: ("American Negro") OR abstract: ("black American") OR abstract: ("black or African American"))) AND ((title: (Patients) OR title: (Patient) OR title: (Clients) OR title: (Client)) OR (abstract: (Patients) OR abstract: (Patient) OR abstract: (Clients) OR abstract: (Client))) AND ((IndexTermsFilt: ("Mental Disorders")) OR (title: ("Mental Disorders") OR title: ("Mental Disorder") OR title: ("Psychiatric Disorders") OR title: ("Psychiatric Disorder") OR title: ("Psychiatric Diseases") OR title: ("Psychiatric Disease") OR title: ("Psychiatric Illnesses") OR title: ("Psychiatric Illness") OR title: ("Mental Illness") OR title: ("Mental Illnesses") OR title: ("Behavior Disorders") OR title: ("Psychiatric Diagnosis") OR title: ("Severe Mental Disorder") OR title: ("Severe Mental Disorders")) OR (abstract: ("Mental Disorders") OR abstract: ("Mental Disorder") OR abstract: ("Psychiatric Disorders") OR abstract: ("Psychiatric Disorder") OR abstract: ("Psychiatric Diseases") OR abstract: ("Psychiatric Disease") OR abstract: ("Psychiatric Illnesses") OR abstract: ("Psychiatric Illness") OR abstract: ("Mental Illness") OR abstract: ("Mental Illnesses") OR abstract: ("Behavior Disorders") OR abstract: ("Psychiatric Diagnosis") OR abstract: ("Severe Mental Disorder") OR abstract: ("Severe Mental Disorders")) OR (title: ("mental disease")) OR (abstract: ("mental disease"))) AND ((title: (Diagnosis) OR title: (Diagnoses) OR title: (Diagnose) OR title: ("Diagnoses and Examinations") OR title: ("Diagnoses and Examination") OR title: ("Examination and Diagnoses") OR title: ("Examinations and Diagnoses")) OR (abstract: (Diagnosis) OR abstract: (Diagnoses) OR abstract: (Diagnose) OR abstract: ("Diagnoses and Examinations") OR abstract: ("Diagnoses and Examination") OR abstract: ("Examination and Diagnoses") OR abstract: ("Examinations and Diagnoses")) OR (title: ("diagnostic screening") OR title: (diagnostics) OR title: ("disease diagnosis")) OR (abstract: ("diagnostic screening") OR abstract: (diagnostics) OR abstract: ("disease diagnosis"))) AND ((IndexTermsFilt: ("International Classification of Diseases")) OR (title: ("International Classification of Diseases") OR title: ("International Classification of Disease") OR title: ("Disease International Classification") OR title: ("International Statistical Classification of Diseases and Related Health Problems") OR title: ("ICD Codes") OR title: ("ICD Code") OR title: ("International Classification of Disease Codes") OR title: (ICD-10) OR title: (ICD-9) OR title: (ICD-9-CM) OR title: (ICD-10-CM) OR title: (ICD-11) OR title: ("ICD-10 Procedure Coding System") OR title: ("ICD 10 Procedure Coding System") OR title: (ICD-10-PCS)) OR (abstract: ("International Classification of Diseases") OR abstract: ("International Classification of Disease") OR abstract: ("Disease International Classification") OR abstract: ("International Statistical Classification of Diseases and Related Health Problems") OR abstract: ("ICD Codes") OR abstract: ("ICD Code") OR abstract: ("International Classification of Disease Codes") OR abstract: (ICD-10) OR abstract: (ICD-9) OR abstract: (ICD-9-CM) OR abstract: (ICD-10-CM) OR abstract: (ICD-11) OR abstract: ("ICD-10 Procedure Coding System") OR abstract: ("ICD 10 Procedure Coding System") OR abstract: (ICD-10-PCS)) OR (title: ("Diagnostic and Statistical Manual of Mental Disorders") OR title: (DSM-IV) OR title: (DSM-II) OR title: (DSM-III) OR title: (DSM-V)) OR (abstract: ("Diagnostic and Statistical Manual of Mental Disorders") OR abstract: (DSM-IV) OR abstract: (DSM-II) OR abstract: (DSM-III) OR abstract: (DSM-V)) OR (title: ("DSM classification")) OR (abstract: ("DSM classification")) OR (title: ("Psychiatric Status Rating Scales") OR title: ("Katz Adjustment Scales") OR title: ("Wittenborn Scales") OR title: ("Edinburgh Postnatal Depression Scale") OR title: ("Mini International Neuropsychiatric Interview")) OR (abstract: ("Psychiatric Status Rating Scales") OR abstract: ("Katz Adjustment Scales") OR abstract: ("Wittenborn Scales") OR abstract: ("Edinburgh Postnatal Depression Scale") OR abstract: ("Mini International Neuropsychiatric Interview")) OR (title: ("psychiatric rating scale")) OR (abstract: ("psychiatric rating scale")) OR (title: ("International Classification of Diseases 10th Revision") OR title: ("International Classification of Diseases Tenth Revision")) OR (abstract: ("International Classification of Diseases 10th Revision") OR abstract: ("International Classification of Diseases Tenth Revision"))) | **02/04/2025**  **25** | **27/02/2026**  **25** |
| **ProQuest Dissertations & Theses Global** | (Adult OR Adults OR "Young Adult" OR "Young Adults" OR "Middle Aged" OR "Middle Age" OR Aged OR Elderly OR "Older Adults" OR "Older people" OR "Older person" OR Senior OR "Oldest adults" OR Elders OR "Very elderly" OR "Aged, 80 and over" OR "Oldest Old" OR Centenarians OR Centenarian OR Nonagenarians OR Nonagenarian OR Octogenarians OR Octogenarian) AND (title("Black People" OR "Black Peoples" OR "People, Black" OR "Black Person" OR "Black Persons" OR "Negroid Race" OR "Negroid Races" OR "Race, Negroid" OR "African Continental Ancestry Group") OR abstract("Black People" OR "Black Peoples" OR "People, Black" OR "Black Person" OR "Black Persons" OR "Negroid Race" OR "Negroid Races" OR "Race, Negroid" OR "African Continental Ancestry Group") OR title("Black Population" OR "Black man" OR "Black race" OR Negroid OR "Negroid race" OR Negroids) OR abstract("Black Population" OR "Black man" OR "Black race" OR Negroid OR "Negroid race" OR Negroids) OR title("Black or African American" OR "Black Americans" OR "American, Black" OR "Black American" OR Blacks OR Negroes OR Negro OR "African Americans" OR "African American" OR "American, African" OR "Afro-American" OR "Afro American" OR "Afro-Americans" OR "Afro Americans" OR "African-Americans" OR "African-American") OR abstract("Black or African American" OR "Black Americans" OR "American, Black" OR "Black American" OR Blacks OR Negroes OR Negro OR "African Americans" OR "African American" OR "American, African" OR "Afro-American" OR "Afro American" OR "Afro-Americans" OR "Afro Americans" OR "African-Americans" OR "African-American") OR abstract("American blacks" OR "American Negro" OR "black American" OR "black or African American") OR title("American blacks" OR "American Negro" OR "black American" OR "black or African American")) AND (abstract(Patients OR Patient OR Clients OR Client) OR title(Patients OR Patient OR Clients OR Client)) AND (abstract("Mental Disorders" OR "Mental Disorder" OR "Psychiatric Disorders" OR "Psychiatric Disorder" OR "Psychiatric Diseases" OR "Psychiatric Disease" OR "Psychiatric Illnesses" OR "Psychiatric Illness" OR "Mental Illness" OR "Mental Illnesses" OR "Behavior Disorders" OR "Psychiatric Diagnosis" OR "Severe Mental Disorder" OR "Severe Mental Disorders") OR title("Mental Disorders" OR "Mental Disorder" OR "Psychiatric Disorders" OR "Psychiatric Disorder" OR "Psychiatric Diseases" OR "Psychiatric Disease" OR "Psychiatric Illnesses" OR "Psychiatric Illness" OR "Mental Illness" OR "Mental Illnesses" OR "Behavior Disorders" OR "Psychiatric Diagnosis" OR "Severe Mental Disorder" OR "Severe Mental Disorders") OR title("mental disease") OR abstract("mental disease")) AND (abstract(Diagnosis OR Diagnoses OR Diagnose OR "Diagnoses and Examinations" OR "Diagnoses and Examination" OR "Examination and Diagnoses" OR "Examinations and Diagnoses") OR title(Diagnosis OR Diagnoses OR Diagnose OR "Diagnoses and Examinations" OR "Diagnoses and Examination" OR "Examination and Diagnoses" OR "Examinations and Diagnoses") OR title("diagnostic screening" OR diagnostics OR "disease diagnosis") OR abstract("diagnostic screening" OR diagnostics OR "disease diagnosis")) AND (abstract("Diagnostic and Statistical Manual of Mental Disorders" OR DSM-IV OR DSM-II OR DSM-III OR DSM-V OR "DSM classification") OR title("Diagnostic and Statistical Manual of Mental Disorders" OR DSM-IV OR DSM-II OR DSM-III OR DSM-V OR "DSM classification") OR title("International Classification of Diseases" OR "International Classification of Disease" OR "Disease International Classification" OR "International Statistical Classification of Diseases and Related Health Problems" OR "ICD Codes" OR "ICD Code" OR "International Classification of Disease Codes" OR ICD-10 OR ICD-9 OR ICD-9-CM OR ICD-10-CM OR ICD-11 OR "ICD-10 Procedure Coding System" OR "ICD 10 Procedure Coding System" OR ICD-10-PCS OR "International Classification of Diseases 10th Revision" OR "International Classification of Diseases Tenth Revision") OR abstract("International Classification of Diseases" OR "International Classification of Disease" OR "Disease International Classification" OR "International Statistical Classification of Diseases and Related Health Problems" OR "ICD Codes" OR "ICD Code" OR "International Classification of Disease Codes" OR ICD-10 OR ICD-9 OR ICD-9-CM OR ICD-10-CM OR ICD-11 OR "ICD-10 Procedure Coding System" OR "ICD 10 Procedure Coding System" OR ICD-10-PCS OR "International Classification of Diseases 10th Revision" OR "International Classification of Diseases Tenth Revision") OR abstract("Psychiatric Status Rating Scales" OR "Katz Adjustment Scales" OR "Wittenborn Scales" OR "Edinburgh Postnatal Depression Scale" OR "Mini International Neuropsychiatric Interview" OR "psychiatric rating scale") OR title("Psychiatric Status Rating Scales" OR "Katz Adjustment Scales" OR "Wittenborn Scales" OR "Edinburgh Postnatal Depression Scale" OR "Mini International Neuropsychiatric Interview" OR "psychiatric rating scale"))  <https://www.proquest.com/search/2841767?accountid=8113> | **03/04/2025**  **07** | **27/02/2026**  **07** |
| **TOTAL** |  | **2.916** | **2.926** |
| **TOTAL DE REFERÊNCIAS EM DUPLICIDADE** |  |  | **842** |
| **TOTAL APÓS EXCLUSÃO DE DUPLICIDADE** |  |  | **2.084** |
