# Supplementary material for: Mental disorders in the Black population: a scoping review
Source: Front Public Health. 2026 Sep 3;14:1903601. doi: 10.3389/fpubh.2026.1903601 (PMC13581542; doi:10.3389/fpubh.2026.1903601)
Supplement: Supplementary file 3 [file Table_3.DOCX]

***Supplementary Material***

# 1 Appendix C. Data Extraction Instrument

| **Scoping Review Details** | |
| --- | --- |
| **Scoping Review Title** | Mental Disorders in the Black Population: A Scoping Review |
| **Review Objective(s)** | To map and summarize the available empirical evidence on mental disorders in the Black population. |
| **Review Question(s)** | What evidence is available on the occurrence of mental disorders in the Black population worldwide? |
| **Inclusion/Exclusion Criteria** | Population: Black people; Concept: mental disorders; Context: global; Sources: empirical studies, reviews, and grey literature (theses, dissertations, guidelines). |
| **Population** | Black people, defined by self-identification or by the authors’ classification. No restrictions regarding age, gender, class, educational level, or country. |
| **Concept** | Mental disorders according to the DSM and/or ICD, including clinical manifestations, diagnoses, access to services, associated factors, and psychosocial consequences. |
| **Context** | Any location, health system, or cultural setting, including public, private, community-based, hospital-based, or non-hospital services. |
| **Details and Characteristics of Evidence Sources** | Quantitative, qualitative, and mixed-methods studies, reviews, and grey literature (theses, dissertations, protocols, guidelines). |
| **Citation Details (author, date, title, journal, volume, issue, pages)** | Author, year, title, journal, volume, issue, pages (or equivalent information for theses, reports, and grey literature). |
| **Country** | Country where the study was conducted. |
| **Context** | Study setting, type of service, type of health system, and cultural and social characteristics. |
| **Participants (age/gender and number)** | Age range, gender, sample size, description of the study population, and criteria used for racial definition. |
| **Description of the Mental Disorders Investigated** | Detailed description of the mental disorders addressed in the study. |
| **Diagnostic Classification (DSM, ICD, other)** | Diagnostic classification system used (e.g., DSM-IV, DSM-5, ICD-10, ICD-11, other). |
| **Social harm related to drug criminalization:** | Punitive indicators (imprisonment, fines); social impacts (stigma, unemployment, etc.) |
| **Identified Associated Factors** | Main factors associated with mental disorders, such as social determinants, racism, vulnerability, and racial bias. |
| **Identified Structural or Social Barriers** | Structural, institutional, social, or cultural barriers identified in access, diagnosis, and treatment. |
| **Interventions or Actions Described (if any)** | Description of clinical, community, policy, programmatic, or coping interventions or strategies, if any. |
| **Main Findings** | Main findings reported, including quantitative, qualitative, or mixed-methods data. |
| **Authors’ Conclusions and Notes/Relevance to the Review** | Authors’ conclusions, implications, gaps in the literature, and observations relevant to the review. |
